# The Role of Quercetin, a Flavonoid in the Management of Pathogenesis Through Regulation of Oxidative Stress, Inflammation, and Biological Activities

**DOI:** 10.3390/biom15010151

**Published:** 2025-01-20

**Authors:** Hajed Obaid A. Alharbi, Mohammad Alshebremi, Ali Yousif Babiker, Arshad Husain Rahmani

**Affiliations:** Department of Medical Laboratories, College of Applied Medical Sciences, Qassim University, Buraydah 51452, Saudi Arabia

**Keywords:** quercetin, oxidative stress, inflammation, cancer, apoptosis, synergistic effects, pathogenesis

## Abstract

Quercetin, a flavonoid found in vegetables and fruits, has been extensively studied for its health benefits and disease management. Its role in the prevention of various pathogenesis has been well-documented, primarily through its ability to inhibit oxidative stress, inflammation, and enhance the endogenous antioxidant defense mechanisms. Electronic databases such as Google Scholar, Scopus, PubMed, Medline, and Web of Science were searched for information regarding quercetin and its role in various pathogeneses. The included literature comprised experimental studies, randomized controlled trials, and epidemiological studies related to quercetin, while editorials, case analyses, theses, and letters were excluded. It has been reported to have a wide range of health benefits including hepatoprotective, antidiabetic, anti-obesity, neuroprotective, cardioprotective, wound healing, antimicrobial, and immunomodulatory effects, achieved through the modulation of various biological activities. Additionally, numerous in vitro and in vivo studies have shown that quercetin’s efficacies in cancer management involve inhibiting cell signaling pathways, such as inflammation, cell cycle, and angiogenesis, activating cell signaling pathways including tumor suppressor genes, and inducing apoptosis. This review aims to provide a comprehensive understanding of the health benefits of quercetin in various pathogeneses. Additionally, this review outlines the sources of quercetin, nanoformulations, and its applications in health management, along with key findings from important clinical trial studies. Limited clinical data regarding quercetin’s safety and mechanism of action are available. It is important to conduct more clinical trials to gain a deeper understanding of the disease-preventive potential, mechanisms of action, safety, and optimal therapeutic dosages. Furthermore, more research based on nanoformulations should be performed to minimize/overcome the hindrance associated with bioavailability, rapid degradation, and toxicity.

## 1. Introduction

Natural compounds derived from different sources, such as plants including seeds, flowers, stems, leaves, and animals, have proven their inhibitory potential in diseases. The disease-preventing ability of natural products has been evidenced through antioxidant, anti-inflammatory, hepatoprotective, cardioprotective, neuroprotective, antidiabetic potential, and antimicrobial activities. Moreover, natural products have played a crucial role in drug discovery, particularly in cancer, infectious diseases [1,2], and other therapeutic areas such as cardiovascular diseases and multiple sclerosis [3,4,5]. Herbs and their bioactive ingredients undeniably play a critical activity in disease prevention due to their rich sources of antioxidants [6,7].

Quercetin (Qu), a flavonoid found in vegetables and fruits, has been shown to significantly prevent and treat pathogenesis by reducing oxidative stress, inflammation, and the modulation of biological activities. Previous study results have reported that lipopolysaccharides caused an increase in intracellular reactive oxygen species levels, which diminished after quercetin treatment. Moreover, this compound reduced the inflammatory cytokine levels, which increased meaningfully after lipopolysaccharide exposure [8]. Quercetin reduced the release of IL-1β, PGE2, IL-6, and nitrite in LPS-induced A549 cells. Furthermore, quercetin relieves ROS generation and inhibits cell apoptosis in LPS-induced A549 cells. Quercetin also inhibits LPS-induced NF-κB activation. This study proposed that quercetin reduces LPS-induced inflammation by activating the Nrf2 signaling pathway [8]. Furthermore, it was reported that quercetin improved neurological deficits, pathological features, and infarct volume in MCAO rats and also increased the viability of HT-22 cells exposed to H_2_O_2_ as well as erastin. These outcomes, together with the MDA, GSH, SOD, ROS levels and iron accumulation, have designated that this compound decreases the generation of lipid peroxides and might be involved in the regulation of ferroptosis [9].

Another study investigated whether quercetin promoted normal epithelial regeneration from COPD airway basal cells (BCs) by altering gene expression. Study based on findings concluded that quercetin may improve airway epithelial regeneration through increasing the expression of genes involved in epithelial development/differentiation in COPD [10]. This compound improved the P. aeruginosa-induced lung injury through diminishing the production of and proinflammatory cytokine neutrophil infiltration, which was associated with decreased mortality. Furthermore, the quercetin-treated mice showed reduced phosphorylation levels of PI3K, AKT, IκBα, and NF-κB p65 in lung tissues [11]. A recent study also concluded that quercetin inhibits ferroptosis in the hippocampal neurons through binding to KEAP1 as well as subsequently upregulating the Nrf2/HO-1 signaling pathway [12]. Moreover, it was reported that quercetin not only prevents UVB radiation damage via decreasing the ROS-induced damage to mitochondria, but also by scavenging ROS, along with inhibiting mitochondrial membrane depolarization as well as cell membrane movement [13,14]. This review extensively analyzes the role of quercetin in disease management by reducing and activating various biological activities.

## 2. Methodology

Electronic databases such as Google Scholar, Scopus, PubMed, Medline, and Web of Science were searched for information regarding quercetin and its role in various pathologies. The search utilized keywords including sources of quercetin, antioxidant, antioxidant activity, inflammation, anti-inflammatory potential, antidiabetic properties, hepatoprotective effects, neuroprotective effects, cardioprotective effects, anti-microbial activity, cancer prevention, anticancer research, clinical trials, synergistic effects, and nanoformulation. The included literature comprised experimental studies, randomized controlled trials, and epidemiological studies related to quercetin, while editorials, case analyses, theses, and letters were excluded.

## 3. The Sources of Quercetin and Its Daily Intake

**Figure 1:**
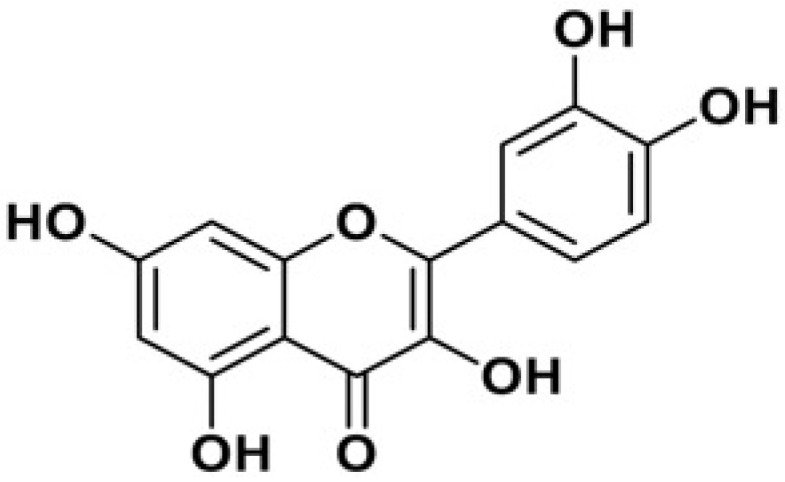
Chemical structure of quercetin (structure was drawn using ChemDraw Professional 15.0).

**Figure 2:**
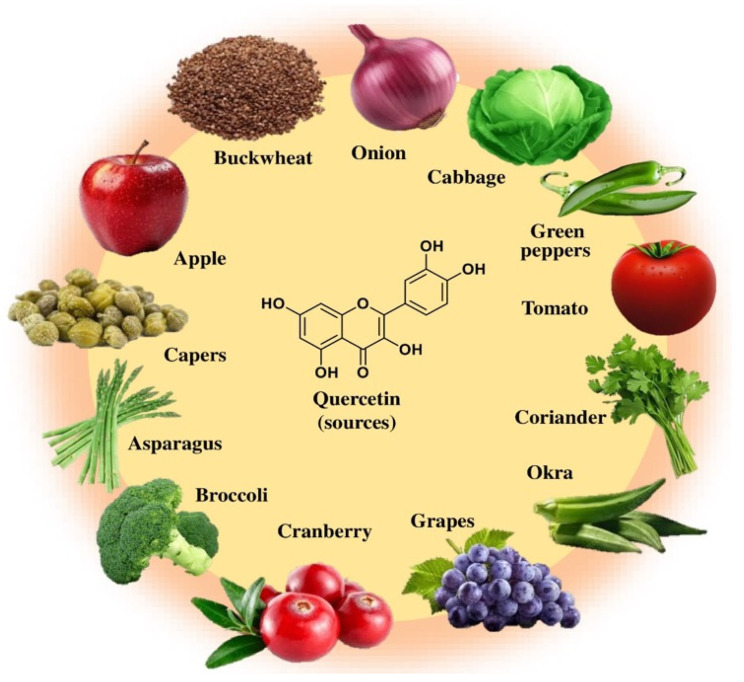
Chief sources of quercetin.

Quercetin (3,5,7,3′,4′-pentahydroxyflavone) (Figure 1) is a flavonoid with a bitter taste. It is found in vegetables and fruits including tomato, green tea, potato, onion, green pepper, apple, parsley, grapes, broccoli, and blueberry (Figure 2).

It has also been stated that quercetin is present in plant species such as tea, coriander, pepper, radish, fennel, and dill [15]. Moreover, other sources of quercetin include vegetables, fruits, berries, nuts, beverages, and other plant-based products [16]. The highest concentration of capers (raw) is 234 mg/100 g of edible portion, while black or green tea has the lowest concentration at 2 mg/100 g of edible portion [17].

The estimated daily intake of flavonoids ranges from 50 to 800 mg, with quercetin accounting for 75% [18]. The intake commonly depends on consuming fruits, vegetables, and tea. The average daily intake of quercetin in Harbin, China is 4.43 mg [18]. The top food sources of quercetin are apple (3.7%), eggplant (2.2%), potato (2.5%), celery (2.2%) as well as Actinidia (1.6%) [19]. In the northern Chinese region of Suihua, the average daily intake of quercetin has been reported as 4.37 mg, where the primary sources of flavonols were apples (7.4%), lettuce (3.8%), potatoes (3.9%), and oranges (3.8%) [20]. In China, the daily intake of quercetin was 20.9 ± 2.32 mg based on the frequency of food consumption reported based on a cross-sectional study of 14,711 adults [21]. In the United States, 13 mg of flavonoid is consumed per day, and it was found that about one-third of it is quercetin [22].

## 4. The Role of Quercetin in Disease Management Through Different Mechanisms

Quercetin, a bioflavonoid, is important in managing diseases by modulating various biological activities. Studies have shown that this compound is involved in various disease processes by inhibiting oxidative stress, inflammation, and modulating cell signaling molecules. The different mechanisms involved in disease management are described below.

### 4.1. Antioxidant Potential

Oxidative stress, inflammation, and apoptosis are importantly interlinked processes that hold immense significance in physiological as well as pathological states [23]. Oxidative stress occurs when an imbalance exists between producing and accumulating reactive oxygen species (ROS) and antioxidants [24]. 

Flavonoids are polyphenols present in vegetables and fruits, and their antioxidant potential is evidence of their potentiality in disease management. Research evidence has shown that dietary polyphenolic compounds are related to the maintenance of human health as well as the prevention of diseases [25].

**Figure 3:**
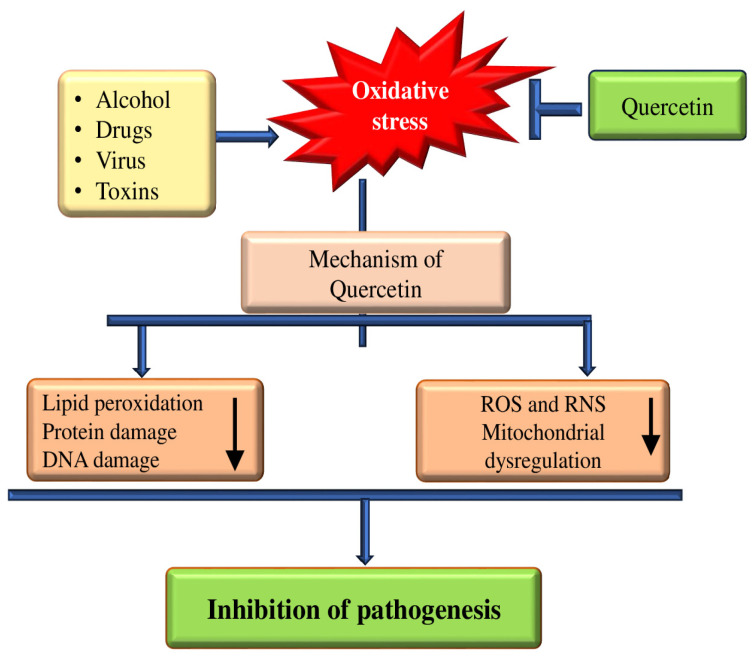
Effects of quercetin in disease prevention through the inhibition of oxidative stress. The downward-pointing arrow shows downregulation.

Regarding oxidative stress or as an antioxidant enzyme activity, the effects of quercetin are presented in Table 1 and Figure 3. The treatment with diquat led to apoptosis in a manner dependent on caspase-3. This was evidenced by increased mitochondrial depolarization, elevated production of reactive oxygen species (ROS), and reduced levels of tight junction proteins. The quercetin administration significantly repealed such adverse effects of diquat. Additional findings indicated that in oxidative stress or antioxidant enzyme activity, the role of the protective effect of quercetin was related to the elevated protein abundance of increased intracellular glutathione (GSH) content [26]. Quercetin supplementation has been found to improve antioxidant defense in the body, as indicated by an increase in total plasma antioxidant capacity [27], and animals exposed to lead and quercetin showed improvement in the albumin, the levels of antioxidant markers, total leucocytic counts, and a decrease in MDA. An exciting finding was noted, as animals exposed to lead and quercetin showed improvement in such parameters, which had moved back toward the control levels [28]. Another study’s results reported that treatment with quercetin suggestively improved arterial blood pressure as well as peripheral vascular resistance. Quercetin provided partial protection to blood glutathione, largely suppressed nitric oxide metabolites, suppressed plasma malondialdehyde levels, and superoxide anion production in the rats with PHZ-induced oxidant stress as well as hemodynamic and disturbance vascular dysfunction [29]. The study was planned to explore the protective effect of prolonged quercetin supplementation on oxidative stress as well as antioxidant enzyme activities in boxers. The study’s findings suggest an increase in the malondialdehyde (MDA) levels of the control group, and there was a significant decrease in the MDA levels of the quercetin group after they were given quercetin [30]. The study aimed to examine quercetin’s effect on tendon adhesion and whether quercetin could inhibit oxidative stress. According to the study’s findings, the extent of glutathione peroxidase and superoxide dismutase in the tendon tissue of the high quercetin group was pointedly greater than those of the low quercetin group and the control group [31]. The results of one study reported that PC-12 cells pretreated with quercetin showed reduced lactate dehydrogenase and increased cell viability when exposed to hydrogen peroxide (H_2_O_2_) [32].

### 4.2. Anti-Inflammatory Potential

Quercetin plays a significant role in managing diseases by regulating oxidative stress and inflammation. Although research has presented important insights into how it functions, the exact pathways through which quercetin exerts its disease-inhibiting effects remain unclear. While there is substantial evidence supporting its involvement in inflammation and oxidative stress, the intricate nature of these pathways designates a need for additional research to fully explain its mechanisms.

Previous studies have outlined how this compound may play a role in disease prevention through its anti-inflammatory and antioxidant properties. Quercetin may hold significant promise in improving atherosclerosis by reducing oxidative stress and inflammatory responses, potentially through the modulation of the AMPK/SIRT1/NF-κB signaling pathway [34]. Under oxidative stress, SIRT1 undergoes deacetylation as the NAD+/NADH ratio rises, which leads to the activation of PGC-1α [35]. This increase in PGC-1α regulates the cellular response to oxidative stress and significantly boosts the gene expression of various antioxidant enzymes such as SOD and GPX1 [36]. Quercetin modifies the mitochondrial metabolism of macrophages via the SIRT1/PGC-1α signaling pathway, thus helping to mitigate oxidative stress damage caused by lipopolysaccharides [37]. Quercetin reduces bovine rumen epithelial cell (BREC) oxidative stress caused by LPS in vitro. Through the interference of the NF-κB signaling pathway, quercetin can reduce the inflammatory response [38]. The anti-inflammatory potential of quercetin in disease management has also been confirmed (Table 2).

The study found that quercetin and galangin effectively reduced the inducible NO synthase, interleukin-6, and the production of nitric oxide (NO). In addition, the natural flavonols inhibited the LPS-induced activation of the extracellular signal-regulated kinase 1/2 (Erk1/2) and c-Jun N-terminal kinase (JNK). The nuclear translocation of nuclear factor-κB (NF-κB) was inhibited, and both flavonols showed a reduction in inflammation, as indicated by ear thickness measurements. Interestingly, when combined, quercetin and galanin were found to be even more effective in decreasing inflammation [39]. An experiment was conducted to assess the ability of quercetin and its human metabolites to counteract the detrimental effects of hyperglycemic or inflammatory conditions on vascular endothelial cells by influencing endothelial cell metabolism. Treating HUVECs with high glucose concentrations significantly increased the glutamate and lactate concentrations. Quercetin prevents glucose-induced increases in adenosine 5′-triphosphate (ATP) and lactate and increases inosine concentrations [40]. An observation has been made regarding glial cells and their response to lipopolysaccharides (LPSs). The mRNA levels of two proinflammatory genes, interleukin 1-α and tumor necrosis factor-α, were significantly lowered when treated with resveratrol or quercetin [41]. Nataliya Chekalina et al. reported that increased levels of TNF-α and IL-1β as well as a moderate increase in IL-10 levels were observed in the serum of patients with coronary artery disease (CAD). Under the effect of quercetin, the levels of TNF-α and IL-1β were decreased, and the IL-10 levels tended to reduce. Additionally, the administration of quercetin reduced the expression of the IkBα gene in contrast to the control group [42]. In a clinical trial that was randomized, double-blind, and placebo-controlled, 50 women diagnosed with rheumatoid arthritis (RA) were separated into two groups. One group was administered a placebo while the other group was given quercetin (500 mg/day) for a duration of 8 weeks. The study revealed that quercetin contributed to the alleviation of morning pain and pain after activity [43].

**Table 2:** Anti-inflammatory potential of quercetin in disease management.

Activity	Types of Study	Outcome	Refs.
Anti-inflammatory	In vitro and in vivo lipopolysaccharide (LPS)-stimulated RAW264.7 macrophages and atopic dermatitis	Quercetin may target NF κB, Erk1/2, and JNK as potential molecular targets in the inflammatory response. The assessment of ear thickness and histological examination indicated that this compound resulted in a reduction in inflammation.	[39]
	In vitro, N9 microglial cells	Resveratrol and quercetin reduce the inflammatory gene. This flavonoid reduced apoptotic neuronal cell death and is a potent anti-inflammatory compound.	[41]
	In vivo, coronary artery disease patient	Quercetin exhibited anti-inflammatory properties in coronary artery disease. Led to a reduction in the transcriptional activity of NF-kB.	[42]
	In vivo, rheumatoid arthritis patients	Supplementation of quercetin per day led to significant improvements in clinical symptoms.	[43]
	In vivo, β-thalassemia major patients	Quercetin reduced high-sensitivity C-reactive protein.	[44]
	In vitro, lipopolysaccharide (LPS)-stimulated RAW264.7 cells	This compound diminished the production of inflammatory markers induced by LPS.	[45]

### 4.3. Antidiabetic Activity

**Figure 4:**
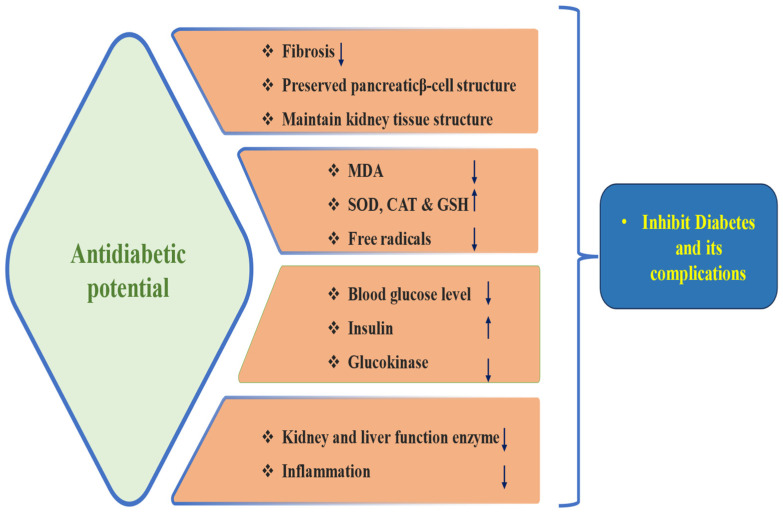
Antidiabetic potential of quercetin. The downward-pointing arrow shows downregulation, whereas the upward arrow indicates upregulation.

Diabetes mellitus is categorized by elevated levels of blood glucose resulting from abnormalities in insulin production or insulin resistance [46]. Individuals may sometimes experience a combination of both factors [46]. It is one of the most abundant metabolic diseases worldwide, associated with approximately 2.8% of the population worldwide. It is predicted to reach 4.4% by 2030, which has already risen to an unprecedented degree of the epidemic [47]. Despite the accessibility of multiple drugs that show antidiabetic potential, effective and safe treatment modules are still needed to overcome adverse complications. Quercetin’s implications as an antidiabetic have also been discussed (Figure 4 and Table 3). The raised serum blood glucose levels, dyslipidemia, and insulin levels in diabetic rats were meaningfully improved by 30 mg/kg quercetin, 10 mg/kg resveratrol, and combined treatments. These compounds intensely prevented oxidative stress as well as tissue injury biomarkers. The compound treatment also preserved the structure of pancreatic β-cells and the activities of hepatic glucose metabolic enzymes from diabetes, and notably, cotreatment with quercetin and resveratrol exhibited the most significant preventive effect on diabetic rats [48]. The antidiabetic potential of quercetin was investigated in streptozotocin-induced diabetics as in normal rats. Glucose tolerance tests of the diabetic animals approached those of normal rats; their plasma triglycerides and cholesterol were reduced, whereas their hepatic glucokinase activity was meaningfully improved after quercetin treatment [49]. Another study reported that quercetin suggestively decreased malondialdehyde and fasting blood sugar levels compared with the diabetic control group based on the results. In contrast, the total antioxidant capacity was enhanced in the quercetin-treated group [50]. The quercetin-treated group of animals displayed a noteworthy decrease in raised blood glucose, NO, and MDA. Additionally, quercetin treatment knowingly increased the pancreatic insulin contents and antioxidant enzyme activities. Moreover, the morphology of the quercetin-treated rats’ pancreases exhibited viable cellularity with distinct beta-cell mass [51]. Quercetin has potential benefits in improving renal function in diabetic nephropathic rats by inhibiting the overexpression of connective tissue growth factor (CTGF) and transforming growth factor beta-1 (TGF-β1) [52]. Another study reported that the oral administration of many doses of quercetin reduced the blood glucose as well as HbA1c levels, increased glycogen synthesis, and reduced α-glucosidase activity and insulin resistance [53]. A pioneer study reported that a substantial decrease in fasting blood glucose level and kidney and liver marker enzymes was noticed in the quercetin-treated group when compared with the diabetic one. Glutathione-S-transferase, glutathione, catalase, and SOD levels were also seen to be enhanced on quercetin supplementation [54].

### 4.4. Hepatoprotective Activity

**Figure 5:**
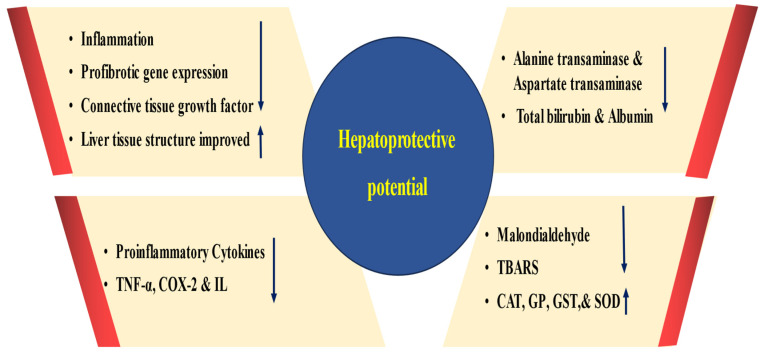
Hepatoprotective potential of quercetin through the modulation of biological activities. The downward-pointing arrow shows downregulation, whereas the upward arrow indicates upregulation.

Quercetin has exhibited remarkable potential in being able to exceptionally manage liver pathogenesis by actively modulating various biological activities. These include a reduction in liver function enzymes, lessening inflammation and oxidative stress, and the preservation of liver and kidney tissues (Figure 5 and Table 4). The hepatoprotective activities of quercetin were investigated, and 2-butoxyethanol caused a significant increase in liver enzymes. In addition, several changes were reported in albumin, total protein, and live tissue alteration. Quercetin with a dose of 50 mg/kg b.w/day and 2-butoxyethanol considerably reduced the activities of albumin, total bilirubin, and liver enzymes [57].

Another study reported that acrylonitrile caused a noteworthy rise in malondialdehyde with a noticeable reduction in glutathione and enzymatic antioxidants, glutathione peroxidase, and superoxide dismutase in the liver. Furthermore, the direct bilirubin, serum alanine aminotransferase, aspartate aminotransferase, and total bilirubin showed a substantial rise in the acrylonitrile-alone treated rats. Pretreatment with quercetin as well as its co-administration with acrylonitrile inhibited acrylonitrile-caused changes in hepatic lipid peroxides as well as enzymatic antioxidants and serum aminotransferases [58].

The potential hepatoprotective effects of quercetin against liver toxicity induced by thioacetamide (TAA) was explored. Thioacetamide administration increased the expression levels of fibrosis-related genes and increased liver indices. Ellagic acid and quercetin administration substantially mitigated hepatic toxicity by reducing liver biomarkers, suppressing the expression of fibrosis-related genes, and enhancing the tissue’s redox status [59]. Cyclosporine A administration decreased the activities of glutathione peroxidase catalase and increased TBARS. A combination of vitamin E and quercetin meaningfully reduced TBARS and increased CAT and GPx in the liver tissue [60], and quercetin-rutinoside (rutin) and its aglycone, quercetin rutin, reduced liver inflammation [61].

An experiment was conducted to determine whether quercetin had any potential benefits in protecting the liver and serving as an antioxidant against acrylamide (ACR)-induced toxicity in rats. Findings revealed that acrylamide caused a reduction in glutathione S-transferase (GST) and increased the 8-OH guanosine level and activity. The administration of quercetin significantly safeguarded hepatic tissue against acrylamide-induced damage, as indicated by the improvement in marker enzymes and DNA damage measured by the comet assay, along with specific indicators of histopathological changes [62]. The study aimed to examine the potential protective effect of quercetin on liver cholestasis and the mechanisms behind it. Quercetin treatment meaningfully lowered the serum levels of the liver function enzymes, TNF-α, and MPO [63].

An orally-given sub-lethal dose of paracetamol (640 mg/kg) caused liver injury in rats, as demonstrated by the important enhancement in the serum levels of aminotransferases (alanine transaminase (ALT) and aspartate transaminase (AST)). The serum enzyme values meaningfully dropped in the pretreatment of rats with either quercetin (10 mg/kg) or caffeic acid (6 mg/kg) [64].

Luis Daniel Hernández-Ortega et al. aimed to explore the effect of quercetin on hepatic stellate cells (HSCs) and the development of hepatic fibrosis. The quercetin treatment group significantly reduced the fibrosis index compared with the control animals. A substantial reduction in hepatic enzymes was observed in the quercetin group. Quercetin enhanced the gene expression and functional activity of antioxidant enzymes and prevented the expression of pro-inflammatory cytokines [65].

**Table 4:** Hepatoprotective potential of quercetin.

Activity	Types of Study	Dose	Mechanism	Outcomes	Refs.
Hepatoprotective potential	In vivo, Sprague-Dawley rat model	50 mg/kg	Albumin and total bilirubin and liver function enzymes ↓	Quercetin exhibited decreased serum enzymes against 2-butoxyethanol-induced oxidative stress.	[57]
	In vivo, albino rat model	70 mg/kg	Hepatic lipid peroxides, serum aminotransferases and bilirubin ↓	Pretreatment with quercetin reduced hepatic lipid peroxides, serum aminotransferases, and bilirubin. It was suggested that quercetin possesses a hepatoprotective effect through its antioxidant activity.	[58]
	In vivo, adult male rat model	100 mg/kg	Liver function enzymes ↓ Oxidative stress ↓ Antioxidant ↑ Matrix metalloproteinase ↓	Quercetin and ellagic acid administration meaningfully reduced hepatic toxicity by reducing liver biomarkers, improving the tissue redox status, and suppressing fibrosis-associated gene expression.	[59]
	In vivo, BALB/cN mice model	10, 50, and 150 mg/kg	Plasma transaminases ↓ Improved the histological signs of liver damage.	Plasma transaminase activity was decreased by quercetin. Quercetin improved the histological signs and lessened liver inflammation.	[61]
	In vivo, male Wistar rat model	10 mg/kg	Glutathione S-transferase activity ↑ 8-OHdG levels ↓ Liver tissue architecture maintenance.	This compound protects the liver against DNA damage, potentially ameliorating changes in rat livers.	[62]
	In vivo, rat model	50 mg/kg	Liver function enzymes and inflammation ↓ Antioxidant enzyme ↑	Quercetin liver injury and decreased liver oxidative stress, inflammation, and fibrosis.	[63]
	In vivo, rat model	10 mg/kg	Liver function enzymes ↓	Quercetin demonstrated hepatoprotective activity, possibly through various mechanisms.	[64]
	In vivo, rat model	100 mg/kg	Fibrosis index and hepatic enzymes ↓ Expression of pro-fibrotic genes, collagen 1α, and connective tissue growth factor ↓	Quercetin reduces oxidative stress and inflammation and inhibits fibrosis by inducing HSC apoptosis and activating MMPs.	[65]

### 4.5. Neuroprotective Effects

Neurodegeneration has been recognized as the significant pathological variation in the majority of brain-related disorders [66]. Herbal medicine and nutraceuticals have been recognized as essential and valuable sources in preventing neurological disorders, rather than their treatment [67]. In fact, in numerous experimental models of neurological diseases, phytoconstituents have reportedly been revealed to have modulatory effects on the nervous system [68].

**Figure 6:**
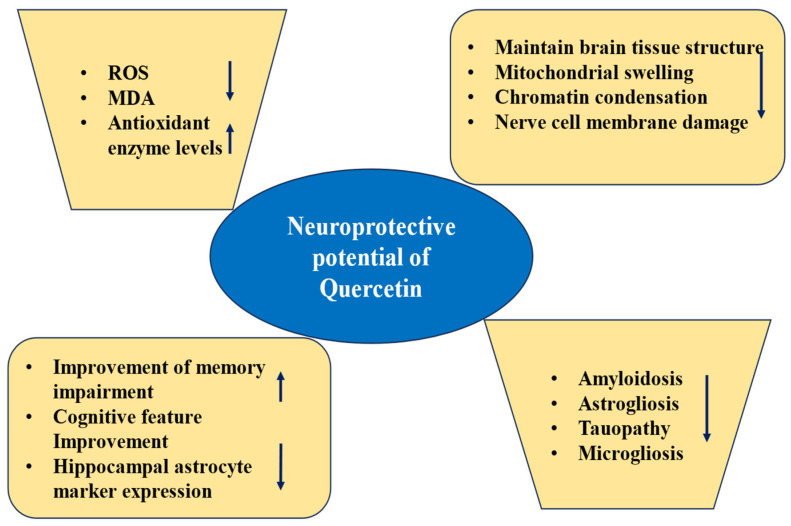
Neuroprotective potential of quercetin. The downward-pointing arrow shows downregulation, whereas the upward arrow indicates upregulation.

The neuroprotective effect of quercetin has been confirmed (Figure 6 and Table 5). One study examined the neuroprotective effect, and quercetin reduced extracellular β-amyloidosis, astrogliosis, tauopathy, and microgliosis in the hippocampus as well as e amygdala. Additionally, based on the elevated plus maze test, it was reported that quercetin induced improved performance on spatial memory tasks and learning as well as more significant risk assessment behavior [69]. Another study reported that quercetin administration reduced aluminum-induced oxidative stress (increased mitochondrial superoxide dismutase and decreased ROS production activity). Moreover, quercetin inhibits aluminum-induced cyt-c translocation, downregulates Bax and p53, upregulates Bcl-2 and caspase-3 activation, and decreases DNA fragmentation [70]. Another experiment was undertaken to investigate the protective effects of quercetin on neurodegeneration. To measure the protective effects, PC12 cells were preincubated with quercetin before H_2_O_2_ treatment, and the cell viability was shown to be improved with quercetin [71].

Quercetin meaningfully increases apoE levels by preventing apoE degradation in immortalized astrocytes. Significantly, it was reported that the oral administration of this compound significantly enhanced brain apoE and decreased insoluble Aβ levels in the cortex of 5xFAD amyloid model mice [72]. The oral administration of nano-encapsulated quercetin improved the memory impairments and cognition features of SAMP8 mice. These notes appeared linked with reduced hippocampal astrocyte marker GFAP expression [73].

### 4.6. Cardioprotective Effects

**Figure 7:**
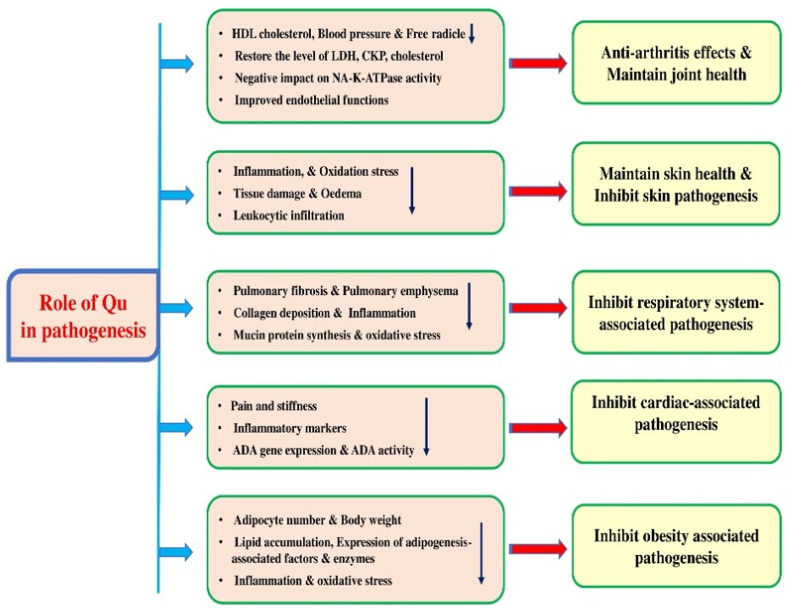
Role of quercetin in different pathogenesis. The downward-pointing arrow shows downregulation.

Quercetin intake has been confirmed to show a significant positive effect in preventing damage produced by oxidative stress. The beneficial effects of quercetin have been chiefly associated with their strong antioxidant and anti-inflammatory activity (Figure 7 and Table 6).

The study aimed to examine the effects of quercetin on lipid metabolism, blood pressure, inflammation, markers of oxidative stress, and body composition. The mean fasting plasma quercetin concentrations increased during quercetin treatment. Quercetin meaningfully reduced the plasma concentrations of atherogenic oxidized LDL compared with the placebo [74].

In spontaneously hypertensive rats (SHRs), quercetin enhanced endothelium-dependent aortic vasodilation and decreased the increase in blood pressure and heart rate induced by acetylcholine. However, quercetin did not affect aortic thromboxane B2 and endothelium-dependent vasoconstriction production. Compared with WKY, SHR displayed downregulated caveolin-1 expression and increased NADPH-induced superoxide production, whereas eNOS activity was reduced. Chronic quercetin treatment inhibited all of these changes in the SHRs [75] (Table 6).

Quercetin had a negative impact on Na,K-ATPase activity as it induced a decrease in the presence of all ATP and Na(+) concentrations that were tested. Furthermore, there appears to have been a decrease in affinity to sodium, as indicated by an increase in the K(Na) value of 22% and 31% in the normotensive and hypertensive groups, respectively [76] (Table 6).

The effect of quercetin on the endothelial function of spontaneously hypertensive rats (SHRs) was examined. Quercetin decreased blood pressure and improved endothelial function in the SHRs. Endothelial autophagy was increased in the early treatment phase and reduced in the late phase of treatment. This compound encouraged autophagy in cultured endothelial cells under oxidative stress and normal conditions. Furthermore, it was noted that the pharmacological prevention of autophagy aggravated endothelial dysfunction in quercetin-treated endothelial cells under oxidative stress, reducing the antihypertensive and endothelial protective effects of quercetin in spontaneously hypertensive rats [77] (Table 6). The role of quercetin in the reduction in blood pressure was examined. Quercetin was added to drinking water in treatment groups at 10, 30, and 60 mg/doses and quercetin resisted the increase in blood pressure [78]. A critical study reported that quercetin treatment only slightly reduced systolic blood pressure. The cross-sectional area as well as the number of vascular smooth muscle cells meaningfully increased in the aortas of hypertensive rats, and quercetin decreased them. Quercetin also increased the activity of gelatinases in situ and decreased the ROS levels [79].

### 4.7. Renoprotective Effects

The renoprotective effects of quercetin have been reported through different mechanisms including as an antioxidant and the maintenance of kidney tissue architecture (Figure 7 and Table 6). The renoprotective effects of quercetin in the animal model of diabetic nephropathy (DN) were explored. Quercetin treatment positively reduced polyuria and glycemia by about 45% and 35%, respectively. Moreover, it abolished hypertriglyceridemia and significantly impacted renal function. Additionally, it appears to have improved structural changes in the kidney including glomerulosclerosis [80] (Table 6).

In the cultured renal tubular cell renal ischemia/reperfusion (I/R) model, quercetin showed a therapeutic implication in reducing cell injury, activating autophagy, and up-regulating the AMPK phosphorylation during I/R. In the I/R mouse model, quercetin altered the renal histological score and decreased the increased serum creatinine level [81] (Table 6). Another study reported that the GSH levels were suggestively improved with quercetin treatment in the I/R group. Histopathological findings, NF-κB, and eNOS expression levels were significantly decreased, and the number of apoptotic and p53-positive cells in the quercetin treatment group was compared with the I/R group [82]. The study was conducted to explore the effect of quercetin, which has antioxidant potential, on Fe-NTA-induced nephrotoxicity in rats. Pretreatment of animals with quercetin half an hour before Fe-NTA administration reduced elevated TBARS and renal dysfunction and restored the depleted renal antioxidant enzymes [83]. A noteworthy finding was made as the administration of quercetin resulted in a reduction in Cd-induced biochemical changes in renal tissue, serum, and urine. Additionally, this compound improved the pathological effects induced by Cd compared with the group treated only with Cd [84].

This study investigated the renoprotective capabilities of quercetin and revealed that cisplatin initiates oxidative stress in renal tissue. Quercetin was able to counteract cisplatin-induced oxidative stress by lowering the levels of free radicals. It also led to the upregulation of antioxidant gene expression and increased antioxidant enzyme activity [85] (Table 6).

### 4.8. Anti-Obesity Effects

Obesity has become one of the most dominant health complications worldwide. It represents a chief risk factor for the development of chronic diseases including type 2 diabetes mellitus [86].

Quercetin and red onion extract (ROE) ameliorated HFD-induced decreases in adipocyte number, increased adipocyte size, and inflammation [87]. The study examined the potential of a quercetin-rich supplement to combat obesity in rats induced by a high-fat diet (HFD). Rats consuming a HFD with a quercetin-rich supplement (185 mg/kg rat) exhibited a significant effect in regulating the final body weights, total body fat, liver weights, and serum triglyceride levels, comparable to rats consuming a regular diet. Additionally, histological analysis demonstrated that the quercetin-rich formulation-supplemented groups showed smaller adipocyte sizes and much less lipid accumulation [88] (Table 6), and quercetin decreased the HFD-induced body weight gain and improved the glucose intolerance and insulin sensitivity of the mice. Quercetin also reduced macrophage infiltration and mast cells [89] (Table 6).

Another study discussed the anti-obesity effects of quercetin, noting that the exposure of 3T3-L1 preadipocytes to quercetin resulted in reduced adipogenesis and the expression of adipogenesis-associated factors and enzymes [90].

Another study was conducted to evaluate the potential benefits of quercetin-rich onion peel extract (OPE) on anti-differentiation in 3T3-L1 preadipocytes as well as anti-obesity in high-fat-fed rats. It was noticed that the lipid accumulations and TG contents in the 3T3-L1 cells were evidently decreased by the extract. The body weight, retroperitoneal as well as mesenteric fat weights of SD rats were meaningfully decreased in the high-fat (HF) diet +0.72% quercetin-rich onion peel extract group [91]. Study findings revealed that quercetin suggestively improved glucose tolerance, HFD-induced obesity, and gut barrier function while also reducing adipose tissue inflammation [92].

### 4.9. Anti-Arthritis Activities

Rheumatoid arthritis is a persistent autoimmune disorder resulting from epigenetic, genetic, non-genetic, and environmental elements [93]. This process targets cartilage and bone, leading to joint deterioration and impairment [93].

Common anti-inflammatory drugs and corticosteroids are used to manage this pathogenesis, but such treatments show various side effects. In this regard, it has been demonstrated that quercetin shows an implication in managing this pathogenesis (Figure 7 and Table 6). A recent study reported that quercetin showed anti-inflammatory potential in a rat rheumatoid arthritis model. The results indicate that purified quercetin shows promising potential as an anti-inflammatory agent in the treatment of rheumatoid arthritis by inhibiting the activity of the adenosine deaminase enzyme [94]. The role of quercetin supplementation on disease severity, inflammation, and clinical symptoms in women with rheumatoid arthritis was also explored, where quercetin supplementation for eight weeks meaningfully reduced morning pain, early morning stiffness, and after-activity pain [43].

According to recent research, quercetin may inhibit neutrophil infiltration and reduce the plasma levels of inflammatory cytokines. These findings suggest that quercetin could be a viable alternative treatment for RA by inhibiting the activities of neutrophils [95]. The study was planned to inspect the impact of quercetin on fibroblast-like synoviocytes in RA. It was noticed that quercetin prevented the production of inflammatory cytokines and the expression of XIST in RAFLSs induced by TNF-α [96] (Table 6). The study focused on investigating the underlying mechanisms responsible for inducing apoptosis of FLSs (fibroblast-like synoviocytes) in patients with rheumatoid arthritis (RA) using quercetin. The study’s findings show that quercetin indorses RAFLS apoptosis via upregulating lncRNA MALAT1 and that MALAT1 induces apoptosis via inhibiting the initiation of the PI3K/AKT pathway [97]. The results indicate that treating fibroblast-like synoviocytes with quercetin had a substantial inhibitory consequence on both their migration and invasion. A marked reduction in the expression of F-actin further supported this. It was noticed that in the RA tissues, the RNA level of miR-146a was remarkably reduced and this decrease was found to be negatively correlated with the expression of GATA transcription factor 6 (GATA6). However, following treatment with quercetin, the RNA level of miR-146a was noticed to increase, whereas the expression of GATA6 in FLSs was suppressed, proposing that quercetin suppresses migration as well as invasion [98] (Table 6).

### 4.10. Role in Skin Health

In an innovative study, the researchers explored the effects of administering galanin or quercetin orally, either separately or in combination, in a mouse model of atopic dermatitis. Accordingly, it was noted that the two flavonols headed for a reduction in inflammation. At the same time, in combination, they were even more effective. Quercetin and galangin have revealed their potential as AD therapeutic agents, and their combination may be a novel preventive approach for AD [39] (Table 6). Quercetin could significantly decrease the PASI scores and improve histopathology in imiquimod-induced mice. Furthermore, quercetin effectively increased the activities of antioxidant enzymes, attenuated the levels of IL-6, TNF-α, and IL-17 in serum, and decreased the MDA in skin tissue by imiquimod in mice [99] (Table 6). Esposito et al. (2020) designed a novel delivery system for treating skin aging and inflammation using sodium alginate-poly(vinyl) alcohol hybrid hydrogels. The quercetin-loaded hydrogel showed good swelling and viscosity profiles, and the hydrogel retained its antioxidant activity after incorporating quercetin [100]. The in vivo efficacy of the polyphenols (quercetin and resveratrol) in liposomes was examined in a mouse model of skin lesions. The topical administration of prepared liposomes led to a notable amelioration of the tissue damage, with a significant decrease in edema as well as leukocyte infiltration [101]. The potential effects of quercetin on P. acnes-induced inflammatory skin disease in vivo and in vitro was investigated. The results showed that quercetin suppressed the production of pro-inflammatory cytokines. In vivo treatment with quercetin reduced swelling and ear thickness [102]. The protective effect of quercetin against UV-mediated skin aging was demonstrated. Quercetin had inhibitory solid effects on the UV-induced activity of activator protein-1 and nuclear factor-kappa B [103].

### 4.11. Effects in the Respiratory System

**Figure 8:**
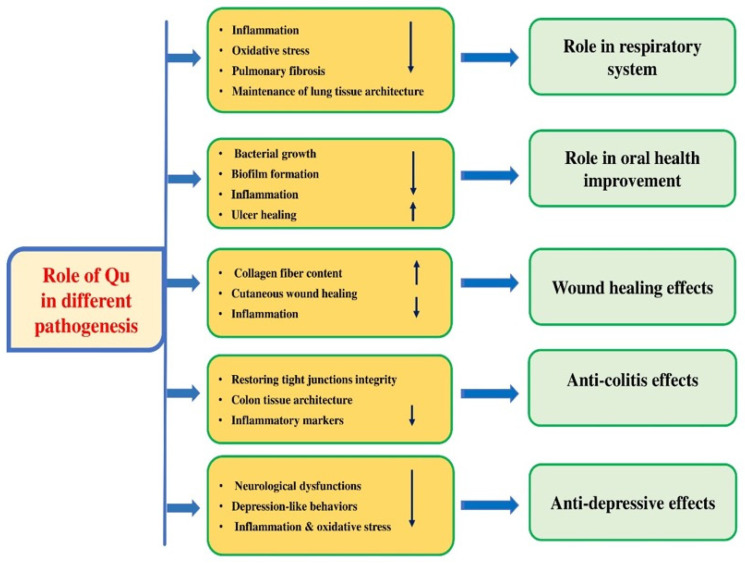
Disease preventive role of quercetin through different mechanisms. The downward-pointing arrow shows downregulation, whereas the upward arrow indicates upregulation.

Respiratory diseases including asthma, COPD, and lung cancer are the leading causes of mortality worldwide. Traditional medicine has a long history of use in treating infectious diseases including viral in addition to respiratory illnesses [104,105]. The role of quercetin in the inhibition of respiratory-associated pathogenesis have been described (Figure 8 and Table 6). A study looked at how quercetin defends the lungs in mice exposed to long-term cigarette smoke. Mice that received quercetin and were exposed to cigarette smoke had less lung damage, fewer inflammatory cells, improved lung structure, lower levels of certain proteins, and less lung enlargement [106] (Table 6). Another study aimed to explore the effect of quercetin on corticosteroid responsiveness in COPD cells. Quercetin treatment activated AMPK and promoted the expression of nuclear factor erythroid 2-related factor 2, effectively reversing corticosteroid insensitivity induced by CSE in U937 cells. In comparison to healthy volunteers, peripheral blood mononuclear cells (PBMCs) from COPD patients exhibited corticosteroid insensitivity, which was restored by quercetin treatment [107].

The study aimed to investigate the potential of quercetin in protecting against mucin expression induced by CS in both in vivo and in vitro experiments. Rats were given an intraperitoneal administration of quercetin or 0.2% Tween aqueous solution before being exposed to CS for 28 days. In vivo-based findings revealed that quercetin pretreatment decreased cigarette smoke-induced goblet cell hyperplasia and inflammation in rat lungs. In vitro, quercetin pretreatment reduced the CSE-induced expression of Muc5ac and NF-κB activation [108] (Table 6). Another study aimed to investigate the therapeutic potential of quercetin for pneumonia. The results revealed that quercetin decreased the release of IL-1β, PGE2, IL-6, and nitrite in LPS-induced A549 cells. Moreover, quercetin relieved ROS generation, inhibited cell apoptosis, and prevented NF-κB activation [8].

In a separate research study, the protective mechanism of quercetin against pulmonary fibrosis was examined. The study involved rats with bleomycin-induced pulmonary fibrosis, where quercetin demonstrated protective effects by decreasing collagen deposition and histological injury as well as alleviating cellular senescence [109]. The study aimed to investigate the relationship between the anti-asthmatic effect of quercetin, periostin, and the downstream molecular pathway of quercetin’s anti-asthmatic effect. More periostin expression was noticed in asthmatic mice, and quercetin reduced the amount of periostin in the bronchoalveolar lavage fluid. A deeper study showed that inhibiting TGF-β1 improved asthmatic symptoms, and quercetin protected asthmatic mice by blocking the TGF-β1/Smad pathway [110].

### 4.12. Effects in Oral Health

Natural bioactive compounds are molecules in plant-based foods that benefit humans [111,112]. Consuming quercetin plays a vital task in inhibiting oral disease development (Figure 8). In an in vivo setting, quercetin supplementation reduced the infiltration of inflammatory cells as well as alveolar bone loss and gingival cytokine expression. In an in vitro environment, quercetin decreased the production of inflammatory cytokines [113]. One study reported that quercetin showed antimicrobial effects and damaged the cell structure, inhibiting gingipain, hemagglutination, hemolytic activities, and biofilm formation [114]. Researchers have also conducted studies to evaluate the effectiveness of Q@HMSNs in preventing dentine erosion and abrasion. Q@HMSNs were effectively synthesized and exhibited negligible toxicity to gingival fibroblasts (HGFs) and human dental pulp stem cells (HDPSCs). Q@HMSNs successfully blocked the dentinal tubules, resulting in a thicker DOM in the Q@HMSN group [115]. Multiplex analysis showed that quercetin increased the proliferation of human oral keratinocytes by upregulating adhesion molecules (Integrin-α6β4). Additionally, the presence of quercetin significantly improved the re-epithelialization rate compared with the control group. Furthermore, quercetin treatment reduced the expression of pro-inflammatory cytokines [116] (Table 6). Quercetin’s effect on inflammatory damage in oral mucosal keratinocytes (hOMK107) induced by lipopolysaccharide (LPS) was assessed, and its underlying mechanism was explored. Quercetin meaningfully improved cell viability as well as apoptosis by reversing the LPS-induced downregulation of Bcl-2 and upregulation of Bax in hOMK107 cells. These findings suggest that quercetin may protect against chronic inflammation-related periodontitis by suppressing the Akt/AMPK/mTOR pathway [117] (Table 6). The study investigated the effectiveness of the topical application of quercetin for treating minor aphthous ulcers. The topical application of quercetin cream to minor mouth ulcers comforted pain and showed complete healing in seven of the Group 2 patients (35%) in 2–4 days, 18 patients (90%) in 4–7 days, and 20 patients (100%) in 7–10 days [118]. In vitro experiments showed that quercetin interfered with the Th1/Th2 balance by acting on IL-6 and IFN-γ to control the immune system in treating oral lichen planus [119].

### 4.13. Wound Healing Effects

The proper treatment of wounds is crucial for maintaining good human health. Delayed healing and prolonged treatment time can lead to increased economic costs [120,121]. Therefore, it is essential to prioritize wound care to ensure the best possible outcomes for everyone involved. Wound healing is a continuous and intricate process at the physio pathological level, involving a multitude of interconnected factors [122]. However, some factors can negatively impact the process such as malnutrition, various drugs, radiation, smoking, and hypoxia [123]. 

Numerous studies have investigated the wound-healing effects of natural products that possess collagen-boosting, anti-inflammatory, and antibacterial properties [124]. A recent study was carried out to assess the healing potential of quercetin on cutaneous wound models in vivo as well as in vitro. A significant finding was noted as this compound promoted both the proliferation and migration of fibroblasts and enhanced the cutaneous wound healing potential in mice. Likewise, the dermal structure in this compound-treated mouse was also restored to normal, and the collagen fiber content increased after administration [125].

Another study was conducted to explore the effects of quercetin gel on a secondary intention wound healing model in Wistar rats, where the wound area reduced from day 0 to day 21 in both groups. Noteworthy differences in wound contraction as well as unhealed wound area were noticed between the seventh and twenty-first days in both groups. The histological findings showed that there was significantly more fibroblast cells present on the seventh, fourteenth, and twenty-first days in the quercetin group compared with the control group. Additionally, in both groups, the inflammatory cell counts decreased significantly from day 0 to day 21. Furthermore, the synthesis of collagen I was at its lowest on day 0, and showed a dramatic increase on all other days in both groups [126] (Table 6). It was observed that wound contraction was the quickest in the Q-HD (high dose) group. Histopathology-based staining showed that collagen deposition and fibroblast distribution in the quercetin-treated groups were meaningfully higher [127] (Table 6).

### 4.14. Anti-Colitis Effects

The role of quercetin as anti-colitis has been evidenced through different mechanism (Figure 8). The effect of quercetin on dextran sodium sulfate caused ulcerative colitis was investigated. Quercetin attenuated dextran sodium sulfate-caused body weight loss, pathological damage to the colon, and colon length shortening. Administration of quercetin modified the gut microbiota composition in mice with DSS-induced colitis and suppressed the growth of harmful bacteria [128]. Another study finding revealed that quercetin and indol-3-carbinol improved clinical symptoms in moderate dextran sodium sulfate colitis, which accords with a suggestively reduced histopathological score [129] (Table 6). Another study confirmed that dietary quercetin at 500 ppm improved DSS-induced colitis, possibly by strengthening antioxidant capacity and intestinal integrity. Based on the results of the colon tissue transcriptome, numerous essential genes were controlled by quercetin [130] (Table 6). An important study was undertaken to examine whether the aryl hydrocarbon receptor (AhR) mediated quercetin’s intestinal barrier repair potential to ameliorate ulcerative colitis. The finding demonstrated that quercetin alleviated colitis in mice via restoring tight junctions. In Caco-2 cells, quercetin increased the expression of the TJ proteins ZO-1 and Claudin1 in a dose-dependent means [131], and other findings showed that quercetin caused a therapeutic potential on *C. rodentium*-induced colitis [132]. One study explored quercetin’s effects on ER stress-mediated apoptosis in an experimental IBD model, where the authors reported that quercetin improved histopathological changes, apoptosis inflammation, and oxidative stress [133].

### 4.15. Role in Inhibition of Platelet Aggregation

Quercetin significantly enhanced the ultrastructural characteristics of platelets and reduced platelet aggregation and thromboxane A2 (TXA2) production while boosting prostacyclin (PGI2) synthesis and the PGI2/TXA2 ratio. The reduction in PC and the rise in TXB2 levels in the plasma suggested that platelets were involved in the arrhythmogenic effects of ischemia and reperfusion [134] (Table 6). In one study, the researchers examined how quercetin inhibited platelet aggregation stimulated by collagen on a molecular level. Quercetin was shown to impact collagen-stimulated whole-cell protein tyrosine phosphorylation and intracellular calcium mobilization in a concentration-dependent manner [135].

In a pilot human dietary intervention study, the association between the consumption of dietary quercetin and platelet function was investigated. The study found that plasma quercetin concentrations peaked at 4.66 µm (±0.77) and 9.72 µm (±1.38) within 30 min of ingesting 150-mg and 300-mg doses of quercetin-4′-O-β-d-glucoside, respectively. These results demonstrated that quercetin was bioavailable and that the plasma concentrations achieved were within the range known to affect platelet function in vitro [136].

### 4.16. Anti-Aging Effects

Aging is one of the chief risk factors for the development of chronic diseases and may increase the risk of death [137,138]. Oxidative stress is a major contributor to cellular damage and plays a key role in the development and progression of age-related pathogenesis. Foods that come from natural sources are packed with several bioactive compounds. These compounds include various compounds, and all are useful for overall health [139]. Quercetin has substantial implications for managing age-related pathogenesis, and research has shown that this compound can alleviate chronic conditions associated with aging by neutralizing reactive oxygen species and its antioxidant properties. A wide range of in vitro and in vivo studies have emphasized quercetin’s ability to combat oxidative stress, establishing it as a promising candidate for alleviating various chronic diseases related to oxidative damage [140,141,142]. Quercetin and its derivative, dihydroquercetin, have shown the capacity to reduce cellular damage caused by oxidative stress by activating the Nrf2-ARE pathway [143]. This process involves the stress-responsive signaling pathways of the extracellular signal-regulated kinase (ERK) as well as the c-Jun N-terminal kinase (JNK), demonstrating that quercetin may have a potential neurohormetic role as a phytochemical [140,144]. Moreover, this compound plays a vital role in regulating oxidative stress and inflammation by modulating the Nrf2 signaling pathway, which is essential for preventing chronic diseases. Its influence on the Nrf2 pathway not only boosts cellular antioxidant defenses, but also affects critical cellular functions like DNA repair and apoptosis. This underscores quercetin’s significance in the management of non-communicable diseases (NCDs) [145].

A total of ten compounds were recognized, and quercetin was examined in detail for its essential effects. Mechanistic studies showed that quercetin improved senescence by restoring heterochromatin architecture and enhancing cell proliferation [146]. Recent study results have shown that at a concentration of 1 mg/L, quercetin significantly extended the average and maximal lifespans of *S. vetulus*, and slightly enhanced the net reproduction rate. Quercetin meaningfully prolonged the average and maximal lifespans of *S. vetulus*, and to some extent, enhanced the net reproduction rate [147].

### 4.17. Anti-Depression Activity

Depression is a common psychological disorder characterized by a range of symptoms including low mood, loss of interest in activities, changes in sleep patterns, appetite changes, difficulty concentrating, and self-evaluation. These symptoms can significantly impact a person’s daily life and well-being [148]. Various types of anti-depressant medicine are used to treat this pathogenesis. However, antidepressant medications may cause adverse effects including sexual dysfunction, cardiovascular problems, and weight gain [149,150]. An important study was performed to examine the role of quercetin on corticosterone (CORT)-induced depression-like behaviors. This study demonstrated that quercetin mitigated depression-like behaviors. Correspondingly, this compound caused antioxidation and anti-inflammatory effects in the hippocampus and prefrontal cortex of CORT-induced mice. Overall, this study revealed that quercetin mitigated CORT-initiated depression-like behaviors, and the mechanism was partly linked to the repression of neuroinflammation as well as oxidative damage [151]. Quercetin or fluoxetine administration increased the body weight of the chronic unpredictable mild stress (CUMS) mice. Behavioral tests showed that the CUMS mice developed a state of depression, while the quercetin or fluoxetine treatment improved their depression-associated behaviors [152] (Table 6). The potential inhibitory role of quercetin on chronic unpredictable stress (CUS)-induced depression was explored. Results revealed that quercetin prevented CUS-induced MDA, and iNOS, and increased the brain tissue levels of Bcl-2 and SOD. Moreover, CUS caused a noteworthy decrease in climbing ability and sucrose consumption and increased animal immobility, which were returned by quercetin [153]. A different research study found that quercetin was able to enhance anxio-depressive-like behavior, reduce oxidative stress of the brain, and prevent excessive corticosterone production in response to adriamycin treatment. Furthermore, quercetin administration partially mitigated adriamycin-induced changes. The study advocates that quercetin may alleviate the neurological and immune system dysfunctions observed in rats treated with adriamycin [154] (Table 6). According to a study by Mehta V. et al., administering quercetin orally (at a dosage of 30 mg/kg) for 21 days resulted in a reduction in anxiety in animals that were exposed to unexpected stress. This suggests that this compound has the potential to improve symptoms of anxiety, depression, and cognitive dysfunction that are induced by stress and prevent neuronal damage [155]. Another study examined the antidepressant-like effects and potential mechanisms of quercetin. The administration of quercetin at a dosage of 50 mg/kg increased sucrose preference in rats suffering from depression. Additionally, this treatment decreased the levels of pro-inflammatory cytokines in the serum of these rats. Furthermore, it was observed that depressed rats had higher levels of iron, calcium, and copper in their serum than the control group. Furthermore, the magnesium, zinc, cobalt, and selenium levels were meaningly decreased in these rats; quercetin treatment restored the levels of these elements [156].

### 4.18. Immunomodulatory Effects

Quercetin is known for its noteworthy activity in inhibiting pathogenesis including modulating the immune system. Studies have shown that quercetin can prevent the increased expression of inflammatory protein and caspase-1 by inhibiting NLRP3 inflammasome stimulation [157]. Quercetin has been found to inhibit the formation of foam cells generated and promote autophagy, which can delay a senescence phenotype, potentially suppressing the progression of atherosclerosis [158]. In addition, quercetin ameliorated inflammation in rheumatoid arthritis mice. This compound reduced the plasma levels of inflammatory cytokines and inhibited neutrophil infiltration [95]. Quercetin effectively reduced Akt phosphorylation, viral endocytosis, and IL-8 responses in airway epithelial cells when used in pretreatment. Interestingly, even when added 6 h after RV infection, quercetin has been observed to decrease the viral load as well as IL-8 and IFN responses [159].

### 4.19. Role in Reproductive System

Research has shown that various natural products offer protective effects against numerous diseases including those that impact the reproductive system [160,161]. Recent studies suggest that consuming plant extracts can enhance semen parameters and androgen status, thus positively affecting male sperm quality [162,163]. A study was conducted to assess the protective role of quercetin on testicular toxicity induced by crude oil vapor (COV). Simultaneous administration of quercetin with COV pointed to a notable reduction in Bax gene expression, enhanced the activities of antioxidant enzymes, and positively impacted reproductive parameters and the expression of the Bcl-2 gene when compared with the COV group [164] (Table 6). Oocytes cultured in a medium added with quercetin showed promising results regarding in vitro maturation and early embryonic development. This led to higher-quality oocytes as well as increased oocyte fertilization and blastocyst-formation rate [165].

Another study demonstrated that quercetin was found to increase the mean blood pressure caused by L-NAME. It was observed that the administration of quercetin not only increased the antioxidant enzyme activities, but also decreased the arginase activity and oxidative stress biomarkers. This compound effectively reversed hypertension-induced impairment by restoring luteinizing hormone, follicle-stimulating hormone, and testosterone levels while also enhancing sperm viability and motility [166] (Table 6).

Hiromi IZAWA et al. reported that diesel exhaust particle (DEP) groups had a substantial impact on the total incidence of sperm abnormalities and daily sperm production in comparison to the vehicle group. However, in mice treated with quercetin plus DEP, the total incidence of sperm abnormalities decreased compared with mice treated with DEP alone. Sertoli cell numbers were reduced in the DEP-treated mice but increased in mice treated with quercetin and onion plus DEP compared with the DEP-treated mice [167]. Exciting study results uncovered the incredible impact of quercetin in increasing birth spacing, leading to a remarkable 60% decrease in the number of litters. This breakthrough has the potential to revolutionize our approach to birth control and reproductive health [168] (Table 6).

Research was conducted to analyze the impact of quercetin on ovarian cell activities. In cultured granulosa cells, the presence of quercetin led to a decrease in the accumulation and transcript levels of PCNA and cyclin B1 while promoting the accumulation of BAX and enhancing the release of T. Additionally, in ovarian follicles, this flavonoid was found to exhibit inhibitory and even reversing effects on FSH. Based on the findings, it can be inferred that quercetin, a natural compound found in plants, can directly decrease the basic functions of ovarian cells [169].

### 4.20. Effects on Bone Health

The role of quercetin on bone-conserving/disease has been widely studied using in vivo and in vitro models. In vivo-based results showed that quercetin effectively increased the bone mineral density and improved the bone biomechanical properties in postmenopausal osteoporosis [170]. In a different study, female C57BL/6J mice that underwent bilateral ovariectomy were given a diet containing 2.5% quercetin for four weeks. The results showed that their total lumbar bone mineral density, cortical thickness, cortical bone area, bone volume/total volume (BV/TV), section modulus, trabecular number, trabecular thickness, and osteoid surface increased. This study provides evidence that quercetin can enhance bone density [171] (Table 6). In vitro studies showed that quercetin meaningfully promoted cell proliferation and the expression of osteogenic and angiogenic factors in a dose-dependent way. Additionally, the nHA bioceramic microspheres could release quercetin sustainably, and quercetin loaded in the nHA bioceramic microspheres could encourage blood vessel formation and new bone formation in vivo. The study findings demonstrated that quercetin could promote angiogenesis and osteogenesis while preventing osteoclastogenesis in vivo and in vitro under osteoporotic conditions [172]. It was noticed that quercetin treatment enhanced the insulin, magnesium, and calcium levels. The results advocate that quercetin treatment may reduce blood glucose and increase plasma insulin, magnesium, and calcium [173] (Table 6).

Wei Liang et al. reported that quercetin (5 mg/kg) displayed a minor effect on diabetic osteopenia, whereas quercetin (30 mg/kg and 50 mg/kg) enhanced the decreased serum osteocalcin in diabetic rats [174].

The study aimed to compare the effectiveness of quercetin with alendronate in preventing glucocorticoid-induced osteoporosis (GIO). This decrease was fully compensated for in groups receiving alendronate plus 50 mg quercetin/kg and quercetin 150 mg/kg. Quercetin markedly raised osteocalcin as a bone formation marker [175].

The treatment with quercetin reduced the number of osteoclasts. In an in vivo mouse calvarial osteolysis model, quercetin was found to inhibit titanium particle-induced osteolysis by preventing the formation of osteoclasts and the expression of ERS-related genes [176].

The flavonoids improved the reduction in bone weight coefficient, the length and diameter of the bone, and the content of bone induced by retinoic acid [177], and a proliferation of osteoblast-like cells was noticeably inhibited upon the exposure of cells to quercetin [178].

### 4.21. Radioprotective Effects

In an experiment, the authors aimed to evaluate the potential radioprotective benefits of quercetin and the ethanolic extract of propolis (EEP) in CBA mice. It was noticed that animals that received pretreatment had less sensitivity to irradiation. Quercetin exhibited better protective effects than EEP in both pretreatment and therapy [179] (Table 6).

The radioprotective effects of quercetin were studied in mice exposed to gamma radiation. It was noticed that quercetin treatment was found to inhibit various free radicals generated in vitro in a concentration-dependent way. This study revealed quercetin’s free radical scavenging action, demonstrating that it may have potential as a radioprotective potential [180] (Table 6). A recent study examined the radioprotective effects of quercetin, a potent free radical scavenger, in protecting human red blood cells and isolated RBC membranes exposed to γ-irradiation-induced oxidative stress. It was noted that quercetin (50 μM) brought the carbonyl level back to normal in γ-irradiated RBC membrane proteins and prevented radiation-induced lipid peroxidation [181].

The radioprotective potential of quercetin against gamma radiation-induced damage in human peripheral blood lymphocytes and plasmid DNA has also been investigated. Radiation exposure displayed noteworthy rises in genetic damage and thiobarbituric acid reactive substances (TBARS), which accompanied an important decrease in the antioxidant status. This flavonoid pretreatment meaningfully reduced the TBARS and genetic damage and enhanced the antioxidant status via its antioxidant potential [182].

**Table 6:** Disease management potential of quercetin via the modulation of various activities.

Activity	Study Types	Animal/Cell Lines	Dose	Outcomes of the Study	Refs.
Cardioprotective	In vivo	Rats	10 mg/kg	High blood pressure and heart rate was decreased quercetin decreased.	[75]
	In vivo	Rats	20 mg/kg	Quercetin demonstrated a detrimental effect on Na, K-ATPase activity by causing a decrease in ATP and Na (+) concentrations.	[76]
	In vivo	Rats	10 mg/kg	Quercetin improved endothelial function and reduced blood pressure. This compound endorsed autophagy.	[77]
Renoprotective	In vivo	Mice	10 g/kg	Quercetin treatment positively reduced polyuria and glycemia. Moreover, it abolished hypertriglyceridemia and impacted renal function. Additionally, improved structural changes in the kidney.	[80]
	In vivo	Mice	5, 10 mg/kg	Creatinine level decreased and histological score improved by quercetin.	[81]
		Rats	50 mg/kg	GSH levels were improved with quercetin treatment. Histopathological observations revealed decreased levels of NF-κB and eNOS expression after quercetin treatment.	[82]
	In vivo	Rats	2 mg/kg	Pretreatment with quercetin reduced elevated TBARS, reduced renal dysfunction. Renal antioxidant enzymes were restored by quercetin treatment.	[83]
		Rats	100 mg/kg	Quercetin could overcome cisplatin-induced oxidative stress. It also induced antioxidant gene expression and antioxidant enzyme activity.	[85]
Anti-obesity	In vivo	Rats	185 mg/kg	Quercetin-rich formulation supplemented groups showed a smaller size of adipocytes and much less lipid accumulation. Decreased serum thiobarbituric acid reactive substances.	[88]
		Mice	0.1% (weight/weight) quercetin	Quercetin attenuated mast cell, macrophage infiltration. Proinflammatory cytokines was lowered by quercetin treatment.	[89]
	In vitro	3T3-L1 preadipocytes	0, 10, 50, or 100 μM	Expression of adipogenesis-related enzymes was decreased by this compound treatment and attenuated adipogenesis. Induction of apoptosis was noticed in the adipocytes with by the treatment of quercetin.	[90]
	In vitro and in vivo	3T3-L1 & Rats	75 and 100 μg/mL and 0.72% OPE	Lipid accumulations and TG contents suppressed. mRNA levels of activating protein downregulated. HF + OPE groups reduced weights in the mesenteric fat pad compared with the HF group	[91]
Anti-arthritis	In vivo	Rats	100 mg/kg	Quercetin caused anti-inflammatory effects, lowering jaw volume, downregulating ADA gene expression, reducing levels of RA cytokines.	[94]
	In vitro	Fibroblast-like synoviocytes	50 nmol/L	Expression of XIST and inflammatory cytokines production prevented by quercetin inhibits	[96]
	In vitro	Fibroblast-like synoviocytes	0, 10, 20, or 30 M	Quercetin treatment caused depressed migration and invasion. GATA6 expression was suppressed and the level of miR-146a increased by quercetin.	[98]
Anti-dermatitis	In vivo	Mice	50 or 100 mg/kg	Galangin and quercetin diminish mast cell infiltration and inflammation in the skin, thus attenuating dermatitis.	[39]
	In vivo	Mice	30, 60, and 120 mg/kg	Anti-psoriasis effects were noted by quercetin via improvement in anti-inflammatory status and as an antioxidant.	[99]
	In vitro, in vivo	HaCaT cells, THP-1, and RAW 264.7, mice	0.01–1 μM and 1 μM and 10 μM	Quercetin suppressed the production of pro-inflammatory as well as output of TLR-2. Quercetin reduced skin inflammation	[102]
Effects in respiratory system	In vivo	Mice	10 mg/kg	Quercetin showed less oxidative damage, improvement in the histological pattern, and reduction in cellular influx. Caused improvement in pulmonary emphysema.	[106]
	In vitro and in vivo	Rats, NCI-H292 cells	25 and 50 mg/kg 5, 10, or 20 μM	Quercetin prevented histopathological changes and attenuated CS-induced inflammatory cell influx. Quercetin attenuated oxidative stress.	[108]
	In vitro	Human alveolar epithelial cell A549	10, 20, and 40 μM	Quercetin attenuated the release of inflammatory markers and nitrite. Quercetin relieves ROS generation and inhibits cell apoptosis.	[8]
Role in oral health	In vitro	Human oral keratinocytes	20 μM	TGF-β3 mRNA and protein levels diminished by quercetin. Expressions of proinflammatory cytokines were downregulated.	[116]
	In vitro	hOMK107 cells	20, 40, and 80 μM	Quercetin caused apoptosis. This flavonoid decreased the inflammatory marker’s production.	[117]
Wound healing effects	In vivo	Rats	Quercetin gel was 5% with a dose of 20 µg	The enhancement in wound healing after quercetin administration noted via decreased inflammatory cells and increased fibroblast cells.	[126]
	In vivo	Rats	10, 20, and 40 mg/mL	The wound contraction was the fastest in the quercetin-high dose group. Histopathological studies revealed that collagen deposition and fibroblast distribution was higher in this compound group.	[127]
Anti-colitis effects	In vivo	Mice	50 mg/kg	Quercetin as well as indol-3-carbinol improved clinical symptoms of colitis. Loss of epithelial integrity and inflammation was decreased by quercetin treatment.	[129]
	In vivo	Mice	500 ppm	Quercetin alleviated colitis and strengthened intestinal integrity and liver antioxidant capacity.	[130]
	In vivo	Mice	25, 50, and 100 mg/kg	Quercetin repaired intestinal barrier dysfunction.	[131]
Anti-platelet activation	In vivo	Rats	5 mg/kg	Quercetin remarkably improved the ultrastructural deviation of platelets.	[134]
Anti-depressant effects	In vivo	Mice	50 mg/kg	QUE or FLX treatment improved depression-associated behaviors.	[152]
	In vivo	Rats	60 mg/kg	Quercetin improved the anxiety-depressive-like behavior.	[154]
Role in reproductive system	In vivo	Rats	50 mg/kg	An increased antioxidant enzyme and decrease in Bax gene expression was noted in the quercetin + crude oil vapor group.	[164]
	In vivo	Rats	50 mg/Kg	Quercetin restored hypertension-induced impairment of hormones. Quercetin improves sperm motility as well as viability.	[166]
	In vivo	Mice	5 mg/Kg	Quercetin increased birth spacing and caused reduction in the number of litters.	[168]
Role in bone health	In vivo	Mice	0.25% (LQ) or 2.5% quercetin	Quercetin inhibits bone loss.	[171]
	In vivo	Rats	15 mg/kg	This compound treatment improves bone biomechanical strength and bone structure.	[173]
	In vivo	Rats	5, 30, and 50 mg/kg	Bone loss was prevented by oral administration of quercetin.	[174]
Radioprotective effects	In vivo	Mice	100 mg/kg	Radioprotective effects of quercetin were noted.	[179]
	In vivo	Mice	20 mg/kg	Quercetin in mitigating radiation-induced oxidative stress and scavenged the radiation-induced free radicals.	[180]

### 4.22. Anticancer Effects

**Figure 9:**
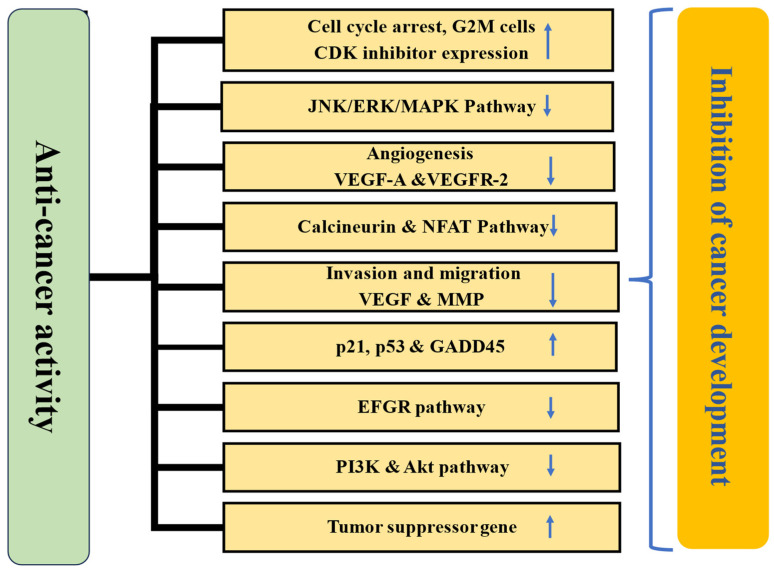
Role of quercetin in cancer prevention through the modulation of cell signaling pathways. The downward-pointing arrow shows downregulation, whereas the upward arrow indicates upregulation.

Despite the progress in various treatment methods, cancer remains a major cause of death worldwide [183,184]. Generally, cancer is defined as a pathological condition marked by unchecked cell growth and proliferation brought on by the accumulation of genetic alterations [185,186]. The development and progression of cancer are predisposed by a range of factors, notably oxidative stress and inflammation. Regulating reactive oxygen species (ROS) levels has shown promising potential as a therapeutic approach, particularly when utilizing the anticancer properties of natural products [187]. Interestingly, several epidemiological studies show a negative relationship between the incidence or progression of cancer with a diet heavy in fruits and vegetables [188,189]. According to epidemiological and laboratory studies, emerging research suggests that a diet rich in flavonoids may lower the cancer risk [190]. Vegetables and fruits are high sources of flavonoids, and flavonoids have shown effects in cancer prevention by modulating cell signaling molecules including the induction of autophagy and the modulation of other cell signaling molecules [191,192,193,194,195,196]. Quercetin acts as a chemopreventive by modulating cell signaling molecules (Figure 9 and Table 7).

A recent study examined quercetin’s effect on cell proliferation as well as on the apoptosis of breast cancer cells. The experiment results indicated that the activity of breast cancer cells diminished as the quantity of quercetin increased, and a concentration-dependent manner of the inhibition of growth was noted. Furthermore, these cells’ nuclear concentration as well as apoptosis also increased with increasing concentrations of quercetin. At high concentrations of quercetin, there was a substantial increase in the expression as well as distribution of PTEN protein [197].

Lich Thi Nguyen et al. reported that quercetin, in a time and dose-dependent manner, reduced breast cancer cell viability. Moreover, quercetin had a dual effect on the cells. On the one hand, quercetin increased FasL mRNA expression and p51, p21 and GADD45 signaling activities. t was also reported that quercetin played a significant role in reducing breast cancer cell viability in time-sed cell apoptosis, and on the other hand, it inhibited cell cycle progression [198]. The study’s findings indicated that quercetin substantially affected multiple apoptosis-related proteins [199].

A study on colorectal cancer cells reported that this flavonoid inhibited the protein expression of NF-κB p65 and VEGF-A, and that quercetin meaningfully prevented VEGFR-2 expression as well as translocation in HUVECs [200]. Another study reported that the colony forming ability in esophageal cancer cell lines was decreased with the administration of quercetin [201].

Study results have also reported that cells treated with quercetin showed an increase in the accumulation of cells in the G2/M phase. Notably, a similar level of G2/M arrest was noticed in T47D cells treated with the combination of quercetin and doxorubicin [202]. Furthermore, in oral squamous cell carcinoma cells, quercetin decreased cell viability and induced G1 cell cycle arrest [203].

### 4.23. Anti-Microbial Activity

**Figure 10:**
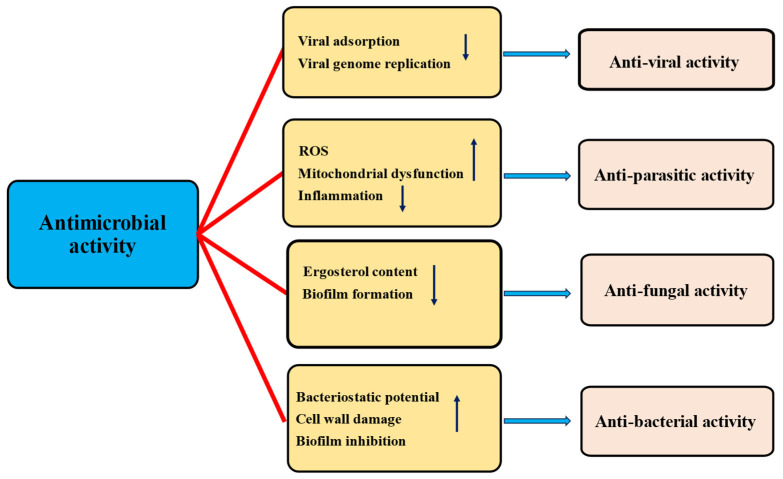
Antimicrobial effects of quercetin. The downward-pointing arrow shows downregulation, whereas the upward arrow indicates upregulation.

The anti-microbial effects of quercetin are evidenced through different mechanisms (Figure 10 and Table 8). Its therapeutic implications as an anti-microbial are described below.

#### 4.23.1. Anti-Bacterial Effects

An investigation was conducted to study how quercetin affected the cecal microbiota of Arbor Acre (AA) broiler chickens in vivo. In addition, the bacteriostatic effect of quercetin as well as its antibacterial mechanism were examined in vitro. Quercetin showed a critical reduction in the copies of Pseudomonas aeruginosa, Staphylococcus aureus, Escherichia coli, Salmonella enterica serotype, and Typhimurium compared with the negative control. Conversely, there was a noteworthy increase in the copies of Bifidobacterium, Lactobacillus, total bacteria [210], and anti-bacterial property of this compound reported by other investigators [211]. It was noted that quercetin showed antibacterial activities against eleven significant oral pathogenic microbes [212].

Drug-resistant *Bacillus subtilis* and *E. coli* growth were hindered by nanoparticles containing quercetin. This was determined through AFM, SEM, and TEM analyses, which showed that the nanoparticles disrupted the cell wall and membrane [213]. It was reported that quercetin binds to the gyrase B of *E. coli* and inhibits the ATPase activity of gyrase B [214], and quercetin was noted to prevent the biofilm production of *Enterococcus faecalis* [215].

#### 4.23.2. Antifungal Activities

The antifungal activity of quercetin was reported, and it was demonstrated that quercetin was effective against T. rubrum. Quercetin decreased the ergosterol content in the two strains, indicating that interference with ergosterol and fatty acid synthesis caused cell membrane disruption [216]. A study was carried out to explore the minimum inhibitory concentration of quercetin for both planktonic and biofilm forms of the Candida parapsilosis complex, where quercetin reduced the metabolic activity of the *C*. *parapsilosis* complex [217].

Cecília Rocha da Silva et al., demonstrated that flavonoids, once combined with fluconazole, displayed effects against strains of *C. tropicalis* resistant to fluconazole, endorsing apoptosis via mitochondrial depolarization, morphological changes, DNA fragmentation, condensation, and the intracellular accumulation of ROS [218].

The study reported that mitochondrial dysfunction activated a decrease in mitochondrial redox levels and led to a disturbance in the mitochondrial antioxidant system. Decreased redox and increased intracellular ROS levels were also noticed, demonstrating overall disruption in antioxidant systems [219].

This flavonoid remarkably inhibited the biofilm of *C. albicans*. Furthermore, oral quercetin displayed a powerful defensive function against *C. albicans* infection in the mouse VVC model [220].

#### 4.23.3. Antiviral Activities

Natural compounds including quercetin play a significant effect in inhibiting pathogenesis through antiviral activity. Quercetin prevented hepatitis C virus (HCV), RNA replication, and inhibited HCV infectious virus production in the HCV contagious cell culture system (HCVcc) [221].

Another study demonstrated that quercetin reduced the generation of HCV-induced reactive oxygen and nitrogen species (ROS/RNS) and reduced lipoperoxidation in replicating cells [222]. Studies have confirmed that quercetin derivatives can potentially prevent viral RNA polymerase. Precisely, quercetin-7-O-glucoside (Q7G) has been found to show robust inhibition activity [223], and research has found that quercetin has promising anti-HBV potential [224].

Quercetin meaningfully reduced the hepatitis B surface antigen (HBsAg), hepatitis B e antigen (HBeAg), HBV genomic DNA, and secretion levels in both cell lines. The findings showed that quercetin inhibited genome replication and HBV antigen secretion in human hepatoma cell lines [225].

To find a possible novel agent, one study examined the effect of quercetin on latent HIV-1 reactivation through an established model of HIV-1 latency. The study’s outcomes established that quercetin efficiently reactivated latent HIV-1 gene expression alone, and led to synergistic reactivation when combined with valproic acid or prostratin [226]. Chenguang Yao et al. reported that quercetin inhibited EV71-mediated cytopathogenic effects, prevented EV71-induced apoptosis, and reduced the EV71 progeny yields [227]. Researchers conducted an in vitro study to examine the impact of quercetin on VP24, which plays a crucial role in virulence by inhibiting the IFN signaling cascade. The study revealed that quercetin successfully suppressed VP24’s anti-IFN function and restored IFN stimulation. Cells infected with wild-type EBOV Makona were treated with QU and Q3G to test whether quercetin might also prevent the replication of EBOV. According to the findings, quercetin effectively prevented viral replication in the HEK293T cells [228].

Quercetin was found to be effective in reducing the infectivity of herpes simplex virus (HSV) in Raw 264.7 cells. It also exhibited inhibitory effects on the expression of HSV proteins (gD, ICP0) and genes (ICP0, UL13, UL52). Remarkably, quercetin was noticed to specifically suppress the expression of TLR-3, which in turn led to the inhibition of inflammatory transcriptional factors including IRF3 and NF-κB [229], and this compound inhibited NS3 protease activity, accordingly decreasing the production of HCV [221].

#### 4.23.4. Anti-Parasitic Effects

An investigation was conducted on the properties of the combination of azithromycin and quercetin on the growth of T. gondii. It was found that the 50% inhibitory concentration for quercetin was 0.50 µM, while azithromycin had an IC50 value of 0.66 µM of interaction with the parasites. Combination testing of quercetin and azithromycin in a ratio of 2:1 exhibited an IC_50_ value of 0.081 µM. Quercetin upregulated reactive oxygen species and caused mitochondrial membrane dysfunction in both intracellular and extracellular T. gondii tachyzoites [230]. Another study investigated the role of quercetin in *L. (V.) braziliensis* infection where quercetin presented antipromastigote (25 ± 0.7 µM) and antiamastigote (IC_50_ of 21 ± 2.5 µM) activities. Oral treatment of quercetin to infected hamsters decreased the lesion thickness and parasite load [231]. Maria Mamani-Matsuda et al. reported that quercetin was revealed to directly induce the death of *Trypanosoma brucei gambiens* [232], and quercetin-loaded phytosomes and quercetin alone had adequate anti-parasitic potential on *P. falciparum* and *L. major* [233].

**Table 8:** Antimicrobial potential of quercetin.

Activity	Study Types	Outcomes	Refs.
Antibacterial effects	In vivo and in vitro	Quercetin decreased the copies of Salmonella enterica serotype, Typhimurium Pseudomonas aeruginosa, Escherichia coli, and Staphylococcus aureus and increased the copies of Bifidobacterium. Quercetin cell walls and membranes damage was noticed by treatment of this compound against *E. coli* and *S. aureus*.	[210]
	In vitro	Quercetin showed antibacterial activity and inhibited bacterial biofilm production.	[211]
	In vitro	Quercetin binds to the gyrase B of *E. coli* and inhibits ATPase activity.	[214]
	In vitro	Quercetin exerts its inhibitory effect by protein translation-elongation, disturbing glycolytic and protein folding pathways.	[215]
Anti-fungal effects	In vitro	Quercetin revealed antifungal action against *T. rubrum*, decreasing the ergosterol levels.	[216]
	In vitro	Quercetin reduced the metabolic activity and biomass of growing biofilms.	[217]
	In vitro	Quercetin-induced yeast apoptosis through mitochondrial dysfunction.	[219]
	In vitro and in vivo	Quercetin prevented biofilm formation and the invasion of *C. albicans* in vitro and in vivo by ameliorating inflammation and protecting the vaginal mucosa.	[220]
Anti-viral effects	In vitro	The inhibitory effect of quercetin on HCV replication was noticed.	[222]
	In vitro	Quercetin showed promising anti-HBV potential.	[224]
	In vitro	Quercetin inhibited HBV antigen secretion as well as genome replication.	[225]
		Quercetin inhibited EV71-mediated cytopathogenic effects	[227]
	In vitro	Quercetin was found to be effective in reducing the infectivity of herpes simplex virus (HSV) in Raw 264.7 cells. It also exhibited prevention properties on the expression of HSV proteins and genes.	[221]
Anti-parasitic effects	In vitro	Quercetin was found to increase the generation of reactive oxygen species and synergize with azithromycin to prevent *T. gondii* growth.	[230]
	In vitro and in vivo	Oral treatment of quercetin to infected hamsters reduced the parasite load and lesion thickness. The antiamastigote activity of the quercetin in vitro is linked, at least in part, with the modulation of production of ROS by macrophages.	[231]
	In Vitro	Quercetin directly promoted the death of *Trypanosoma brucei gambiense*.	[232]

## 5. Synergistic Activities of Quercetin with Other Drugs

**Figure 11:**
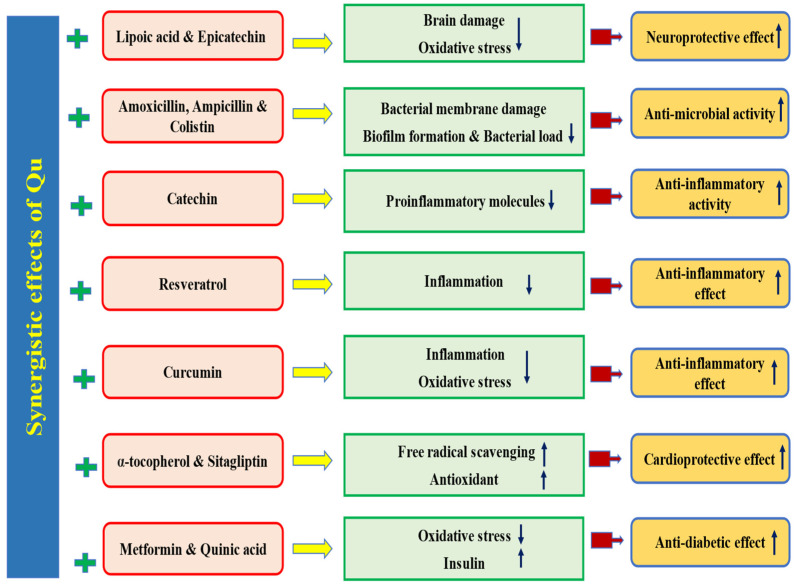
Synergistic effects of quercetin with other compounds/drugs. The downward-pointing arrow shows downregulation, whereas the upward arrow indicates upregulation.

When combined with other drugs or other natural compounds, quercetin can create a synergistic effect that is more effective and comprehensive than that achievable by each compound alone. The research has focused on the impact of this bioactive compound in treating other diseases. It has been described that quercetin enhances bactericidal efficacy and anticancerous potential by modulating the cell signaling pathways. Combining quercetin with other drugs or natural compounds has shown promising results in treating various diseases (Table 9 and Figure 11). The potential role of combining quercetin with colistin, meropenem, and amikacin was assessed. The combinations of quercetin + amikacin and quercetin + colistin exhibited synergistic activity on colistin-resistant *A. baumannii* strains. Moreover, quercetin + colistin and quercetin + amikacin induced membrane damage [234]. The synergetic effect of quercetin with antibiotics (tobramycin, gentamycin, ceftriaxone, levofloxacin, and amikacin) was established. The biofilm cell viability and biofilm formation were considerably affected by quercetin [235]. Quercetin (15 μg/mL) and florfenicol (0.156 to 1.25 μg/mL) reduced the bacterial viability by 16.3–191.4-fold compared with florfenicol alone [236].

A research study assessed the efficacy of flavonoids in combination with antibiotics. The findings revealed that the inhibitory zones expanded when antibiotics facing resistance were combined with flavonoids. Among the flavonoids tested, quercetin demonstrated the highest effectiveness at MIC 260 μg/mL, and the combination of morin, rutin, and quercetin exhibited the most efficient outcome. Combining quercetin and rutin + morin demonstrated synergistic effects when paired with cephradine, amoxicillin, ampicillin, imipenem, ceftriaxone, and methicillin. When used alone, quercetin exhibited an additive effect with ceftriaxone, methicillin, ampicillin, imipenem, and cephradine [237].

The minimum inhibitory concentrations (MICs) against all ARSE strains were as follows: amoxicillin—16 μg/mL, penicillin—200 μg/mL, quercetin—256–384 μg/mL, and kaempferol—>1024 μg/mL. Synergistic effects were shown on amoxicillin + quercetin and penicillin + kaempferol against these strains. The viable count confirmed the synergistic effect of quercetin and amoxicillin. This combination increased the cell membrane permeability and caused significant morphological peptidoglycan as well as cytoplasmic membrane damage [238].

Other findings reported that the combined treatment of quercetin with catechin synergistically reduced the LPS-stimulated increase in some proinflammatory molecules and displayed a powerful inhibitory effect [239]. Moreover, quercetin and resveratrol showed anti-inflammatory properties when administered in isolation or combination (CQR) [240].

The individual and combined impacts of quercetin and curcumin on carrageenan-induced acute inflammation in rats were also examined. Both quercetin and curcumin decreased carrageenin-induced edema and lymphocyte infiltration, and the reduction was even higher in the case of their combination. Furthermore, both flavonoids decreased NO and MDA formation and restored the contents of GSH. Results showed that both quercetin and curcumin moderately decreased inflammation, whereas their combination was more powerful [241]. The pretreatment with curcumin + quercetin synergistically restored the neurohepatic dysfunction and oxidative levels to around normal levels [242].

The combined pretreatment with α-tocopherol and quercetin minimized the alterations in the electrocardiogram and normalized all of the biochemical parameters on isoproterenol-treated myocardial infarcted rats [243]. The cardioprotective effects of quercetin and sitagliptin, each alone or in combination, on doxorubicin (DOX)-induced cardiotoxicity were examined. The combination of sitagliptin and quercetin was more effective than each treatment alone in restoring the level of troponin, lactate dehydrogenase, C-reactive protein, creatine phosphokinase, cholesterol, triglycerides, atherogenic index of plasma and meaningfully enhanced total antioxidant capacity compared to doxorubicin-treated group [244]. The effects of combining epicatechin as well as quercetin on mitochondrial function as well as neuronal survival were evaluated. Relative to the mouse cortical neuron cultures pretreated with either epicatechin or quercetin, epicatechin + quercetin synergistically reduced oxygen-glucose deprivation-induced neuronal cell death. Moreover, epicatechin, quercetin, and epicatechin + quercetin increased the spare respiratory capacity but only epicatechin + quercetin conserved this decisive parameter of neuronal mitochondrial function after oxygen-glucose deprivation [245]. The pretreatment of aluminum chloride exposed rats to either *α*-lipoic acid or quercetin returned altered lipid peroxidation and superoxide dismutase to normal levels. Combined pretreatment of aluminum chloride exposed rats with *α*-lipoic and quercetin acid caused an inclination toward normalization of most of the parameters [246]. The combination of metformin as well as quercetin caused a higher antidiabetic potential than either drug [247], and synergistic effects of quercetin and quinic acid in improving hyperglycemia and hyperlipidemia were noticed [248].

## 6. Bioavailability of Nanoformulated and Quercetin Delivery Methods

The effect of quercetin in various pathogeneses has been validated by in vitro studies. However, when translating these in vitro results to in vivo situations, factors such as bioavailability, metabolism, dosage variations physiological variations, and long-term effects influence the outcomes. Moreover, in vivo research findings support the translation of quercetin’s effects from in vitro to in vivo, but it is critical to undertake a thorough investigation of the factors that influence the outcomes.

One study reported that quercetin was rapidly metabolized and excreted via urine, resulting in very little accumulation in diseased sites [249]. It was shown that healthy volunteers absorbed quercetin (3% and 17%) after receiving 100 milligrams per kilogram of the compound [250]. After a single oral treatment, 93% of this compound was metabolized in rats an hour later, following the administration of 10 mg quercetin per 200 g of body weight [251].

Enhancing the bioavailability of quercetin is crucial to exploring its therapeutic role in disease prevention and treatment. Nanoformulations demonstrate improved efficacy, reduced toxicity, and a prolonged elimination profile, making it an auspicious remedy for therapeutic applications. The bioavailability of various nanoformulations of quercetin significantly exceeds that of natural quercetin due to improved stability and solubility as well as absorption properties. Additionally, nanoformulations can protect quercetin from degradation, permitting it to retain its effectiveness for a longer duration. The pharmacokinetics of a hybrid-hydrogel formulation of quercetin was examined. The pharmacokinetic study confirmed the enhanced pharmacokinetics of formulated QU compared with the unformulated quercetin. The area under the plasma concentration versus time curve estimation displayed an 18.61-fold enhancement in the bioavailability of free forms and was 62.08-fold higher for the total quercetin bioavailability in comparison to an equal dose of unformulated quercetin [252]. The study reported that the self-nanoemulsifying drug delivery system of quercetin meaningfully enhanced its dissolution, displaying its capability to enhance the oral solubility and bioavailability of the drug in the comparison of quercetin [253]. The bioavailability of quercetin-loaded zein nanoparticles in vitro was 5.9%, which is markedly higher than the 1.9% bioavailability of natural quercetin. This shows that the nanoformulation significantly improved the bioavailability of quercetin compared with natural quercetin [254]. The oral absorption of quercetin was substantially enhanced with the LipoMicel delivery system compared with free quercetin. Compared with free quercetin, 8- and 9-times increases in AUC as well as Cmax were obtained with the LipoMicel delivery system, and 10-fold higher quercetin plasma concentrations at 12 h after administration [255]. The oral bioavailability of QUE in the quercetin-loaded poly(lipoic acid) nanoparticles as well as crystalline QUE groups were 29 and 0.19%, correspondingly, suggesting a noteworthy enhancement of oral absorbability, probably due to the improved dissolution and stability property of QUE in the GI tract [256].

**Figure 12:**
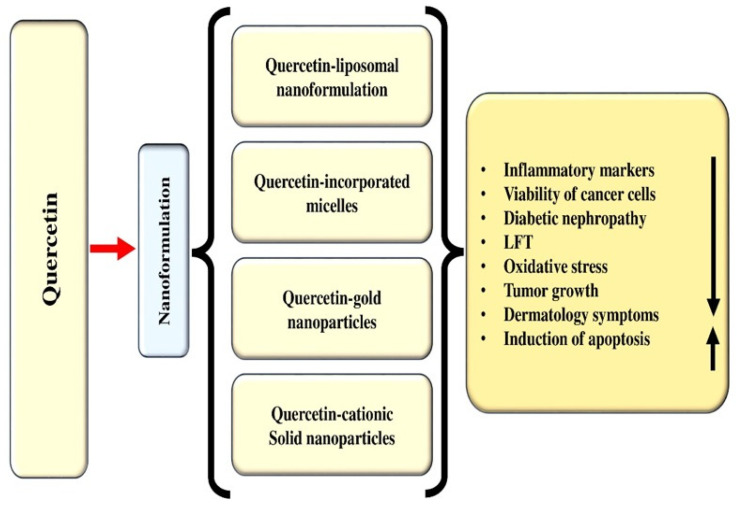
Nanoformulations of quercetin and their role in disease prevention. The downward-pointing arrow shows downregulation, whereas the upward arrow indicates upregulation.

Various delivery methods, such as liposomal nanoparticles, nanoemulsions, micelles, vehicles, nanospheres, and hydrogels, have recently emerged to enhance quercetin’s oral absorption and bioavailability (Table 10 and Figure 12). A study was undertaken to develop a quercetin liposomal formulation, and it was reported that this formulation decreased inflammation markers and improved recovery in a hepatic ischemia and reperfusion injury model [257]. Another study was conducted to load quercetin on nanoliposomes to increase its efficacy against colorectal cancer cells. It was reported that free quercetin and quercetin-loaded liposomes imparted substantial cytotoxicity against cancer cells; the combination form was suggestively more active [258]. The role of quercetin-loaded liposomes against colon cancer cells was also explored. The findings showed that the cells were suggestively more sensitive to this formulation [259].

The purpose of the study was the simultaneous loading of quercetin as well as mint essential oil in phospholipid vesicles precisely tailored to find an antioxidant and antibacterial mouthwash. The vesicles were made by using soy lecithin and Tween 80 as bilayer components, and a mixture of propylene glycol (33%), phosphate buffer solution (33%), and ethanol (33%) as the dispersing phase. All tested formulations were more biocompatible toward epithelial cells as well as proficient in counteracting the oxidative cell damage caused [260]. In a study using a rat model, the role of quercetin liposomes on streptozotocin-induced diabetic nephropathy was examined. The quercetin and liposomal formulation suggestively improved the diabetic nephropathy pathological and biochemistry changes. It was demonstrated that using quercetin liposomes allowed for a decrease in disease symptoms [261].

Quercetin-loaded liposomes were prepared, and it was concluded that these liposomal formulations achieved sustained drug release in wound areas [262]. Quercetin-loaded liposomes modified with galactosylated chitosan improved the livers in mice. More remarkably, they maintained a low content of liver function enzymes and increased levels of GSH [263]. A quercetin-holding liposome-in-gel was prepared, and its antioxidant role was examined. When compared with the untreated animal, the animal treated with this formulation exhibited a substantial decrease in dermatopathologic signs [264]. A study was conducted to develop quercetin-incorporated micelles containing of pluronic P123 and F88. The oxidation resistance of quercetin-incorporating mixed P123/F88 micelles was noticeably higher than that of pure quercetin and effectively inhibited tumor cell growth [265]. Quercetin-loaded polymeric mixed micelles showed a 1.7-times higher cytotoxicity against glioma cells, inhibited migration, and induced apoptosis than the quercetin pure drug [266]. A recent study aimed to examine the quercetin-loaded silver nanoparticles (AgNPs) in hydrogel matrices (QCT-AgNP hydrogel) as synergistic treatment patterns for diabetic wounds. The antimicrobial study demonstrated better therapeutic efficiency of the QCT-AgNP hydrogel in comparison to the marketed gel on *E. coli* and *S. Aureus* [267]. Quercetin-gold nanoparticles were prepared, and their antiproliferative activity was evaluated using human cancer cell lines. Cell culture findings established the potential effectiveness of these nanoformulations against cancer cell lines [268]. A recent study reported that the inflammatory stimulation of BV-2 cells with lipopolysaccharides caused an increased release of proinflammatory prostaglandin, E2, nitric oxide, enhanced COX-2, and inducible NO synthase mRNA, which were inhibited by the pretreatment with gold-quercetin nanoparticles without causing any cytotoxic effects. The study’s findings indicate that the potential of gold-quercetin nanoparticles is superior to quercetin, and that gold-quercetin nanoparticles might be delivered safely against inflammatory neurodegenerative disease through the decrease in acute microglial activation [269]. One study prepared both uncoated and chitosan-coated quercetin-loaded solid lipid nanoparticles and assessed their cytotoxicity on T-24 cells. The cytotoxicity profile results highlighted a clear concentration-dependent toxicity [270]. The protective and antioxidant potential of quercetin-loaded nanostructured lipid carriers (QNLCs) against toxicity was evaluated. It was reported that it significantly restored mitochondrial membrane potential reduction, lipid peroxidation, lysosomal membrane destabilization, and was capable of preventing paraquat (PQ)-caused changes in the expression of the Bax and Bcl2 genes. Furthermore, QNLCs had a meaningfully superior capacity to inhibit PQ-induced toxicity than quercetin itself [271]. The quercetin nanoemulsion decreased the oxidative stress markers and inflammatory biomarkers [272].

For the topical route, it is widely understood that only small neutral lipophilic particles (less than 500 Da) with a logP between 1 and 3 can effectively penetrate the stratum corneum. Conversely, larger or charged molecules need to be delivered using appropriate delivery systems [273]. Traditional topical dosage forms including ointments, creams, gels, and emulsions are the main approaches utilized for formulating quercetin [274]. Advanced delivery systems can ensure enhanced transport of this substance [275]. The study aimed to apply essential oil-based microemulsions to enhance the solubility, photostability, pH stability, and skin permeation of quercetin for topical application. It was reported that the essential oil-based microemulsions exhibited the potential to protect quercetin from degradation. Moreover, the in vitro skin permeation study exhibited that the this microemulsions increase the permeation capacity of quercetin [276]. A quercetin-loaded liposomal hydrogel was made, and the results confirmed accelerated wound-healing with a meaningful reduction in the wound closure time compared with the conventional dosage form [277]. Huang et al. (2022) created a delivery system for QUE utilizing polymeric nanoparticles composed of polyethylene glycol (PEG) combined with polyethyleneimine (PEI) polymers. The findings confirmed that the modifications seen in the serum levels of important biomarkers could be effectively regulated by the PEG–PEI nanoparticles containing QUE. Furthermore, this formulation was shown to reduce inflammation and oxidative stress as well as mitigate the renal damage associated with acute kidney injury [278]. Mok and coworkers suggested a QUE delivery system containing of a methoxy-poly (ethylene glycol)-l-poly(alanine) polymer to inhibit pain and delay the progression of osteoarthritis [279].

**Table 10:** The impact of a quercetin-based nanoformulation on different pathogenic processes.

Nanoformulation	Pathogenesis	Types of Study	Cell Line/Animal	Outcome	Refs.
Quercetin liposomal nanoformulation	Ischemia and reperfusion injury	In vivo	Rat	This formulation prominently decreased inflammation markers and enhanced recovery.	[257]
Quercetin-loaded liposomes	Colorectal cancer	In vitro	SW48 colorectal cancer cells	This formulation decreased the viability of cancer better than the free compound. Furthermore, formulation-induced apoptosis was higher than free quercetin.	[258]
Quercetin-loaded nano-liposomes	Colon cancer	In vitro	HCT-116 p53^+/+^	Loaded liposomes exhibited higher antitumor action compared with free quercetin.	[259]
Quercetin liposomes	Diabetic retinopathy	In vivo	Rat	Quercetin liposomes allow for a decrease in disease symptoms of diabetic nephropathy.	[261]
Quercetin liposomes	Liver injury	In vivo	Mice	This formulation reduced liver function enzymes and oxidative stress,	[263]
Quercetin-containing liposomes-in-gel	Eczema	In vivo	Mice	Formulated liposomes caused a reduction in dermatopathology symptoms	[264]
Quercetin-incorporated micelles	Breast cancer	In vitro	MCF-7 cell line	Formulated micelles effectively in inhibited tumor cell growth.	[265]
Quercetin-loaded polymeric mixed micelles	Brain cancer	In vitro	C6 and U87MG	This formulation caused superior cellular uptake, induced apoptosis, and inhibited migration when tested against pure quercetin.	[266]
Gold-quercetin nanoparticles	Inflammation	In vitro	BV-2 cells	Nanoformulation decreased nitric oxide, proinflammatory prostaglandin, E2, and COX-2.	[269]
Quercetin-loaded cationic solid lipid nanoparticles	Bladder cancer	In vitro	T-24	Nanoformulation caused higher cytotoxicity.	[270]
Quercetin nanoemulsion	Alzheimer’s disease	In vivo	Rat	This compound protected neuronal dysfunction and improved histopathological changes.	[272]

## 7. Quercetin-Based Clinical Trials Studies

Quercetin (Qu) is recognized for its anti-inflammatory activities, and clinical studies have been conducted to examine its role in inflammatory conditions. A clinical trial was conducted that randomly assigned 50 women with rheumatoid arthritis to either receive the treatment or a placebo; participants were divided into a quercetin (500 mg/day) group and a placebo group for eight weeks. The outcomes indicated that quercetin supplementation for eight weeks reduced early morning stiffness, after-activity, and morning pain. The Qu group also showed decreased plasma hs-TNF-α levels compared with the placebo group. Although there were insignificant differences in TJC and swollen joint counts between the two groups, the TJC was suggestively reduced in the Qu group after the intervention [43]. A research study investigated the effects of quercetin on iron overload and inflammation in individuals with thalassemia. The study involved 84 participants who were randomly divided into two groups: one group received a 500 mg/day quercetin tablet, and the other group received a 500 mg/day starch placebo for a duration of 12 weeks. The findings suggested that quercetin may positively impact the iron levels in individuals with major thalassemia, although its influence on inflammation was inconclusive [44]. The study aimed to investigate the impact of quercetin supplementation on the lipid profile, glycemic control, and oxidative stress markers in individuals with type 2 diabetes. Forty-seven participants were randomly assigned to receive either 250 mg of oral quercetin daily or a placebo for a duration of eight weeks. Quercetin supplementation meaningfully increased the overall antioxidant capacity in the intervention group compared with the placebo group and reduced the serum concentration of atherogenic oxidized LDL. Oral supplementation with quercetin improved the antioxidant status in patients with type 2 diabetes [280]. A study was conducted using a randomized, blinded crossover method to investigate the impact of Qu on postprandial hyperglycemia in patients with type 2 diabetes mellitus. The findings indicated that 400 mg of quercetin significantly reduced postprandial hyperglycemia in patients [281]. A research study aimed to investigate the potential therapeutic benefits of quercetin in outpatients experiencing mild to moderate early-stage symptoms of COVID-19. Participants were arbitrarily allocated to receive either the standard of care (SC) along with an oral quercetin supplement or just the standard of care. The results revealed that after one week of treatment, patients in the quercetin group experienced rapid recovery from COVID-19. These findings indicate a potential therapeutic role of quercetin in early-stage COVID-19 including the prompt resolution of acute symptoms [282].

In a 2-week clinical study, 42 COVID-19 outpatients were enrolled, where a total of 21 received the standard of care and 21 received Quercetin Phytosome^®^ (QP) in addition to the standard treatment. After 1 week, sixteen patients in the QP group were tested and shown to be negative, and twelve showed reduced symptoms, while in the SC group, two patients tested negative and four revealed a partial improvement in symptoms. By week 2, five more patients in the QP group tested negative, whereas in the SC group, seventeen tested negative, one tested negative by week three, and regrettably, one patient who continued to be positive passed away by day 20 [283]. A clinical trial was conducted to assess the impact of quercetin at dosages of 500 mg/day and 1000 mg/day compared with a placebo in a group of 1002 patients over a 12-week period. The study revealed that there were no statistically significant differences in the rates of upper respiratory tract infections between the quercetin and placebo groups [284]. The efficacy of quercetin in halting the advancement of the disease into a critical phase and decreasing the levels of inflammatory markers associated with SARS-CoV-2 pathogenesis was analyzed. In an open-label clinical trial, 60 severe cases were randomly assigned to either the control or intervention groups. Quercetin was safe as well as effective in decreasing the levels of LDH, ALP, and q-CRP, which are critical markers involved in the severity of COVID-19 [275,285]. A meta-analysis was conducted to evaluate the potential impact of quercetin on weight loss using data from nine randomized controlled clinical trials with 525 participants. The analysis found that daily quercetin supplementation did not have a significant effect on body weight, waist circumference, body mass index, and waist-to-hip ratio. In conclusion, the current evidence does not support the idea that quercetin intake has a noticeable positive effect on weight loss [286] Supplementation with quercetin-rich onion peel extract (OPE) led to a significant reduction in body weight and body fat percentage. These effects were not observed in the control group. Both groups experienced decreases in the blood glucose and leptin levels. The supplementation of quercetin-rich OPE resulted in changes to the body composition of overweight and obese individuals [287]. Research was carried out to examine how quercetin supplementation affects the blood pressure of individuals with hypertension. The main objective of the study was to establish a potential connection between the antihypertensive impact of quercetin and the reduction in systemic oxidant stress. The trial involved both male and female participants diagnosed with either prehypertension or stage 1 hypertension, and was conducted as a randomized, double-blind, placebo-controlled trial. The results of the study showed that Qu supplementation did not affect blood pressure in prehypertensive patients. However, stage 1 hypertensive patients experienced reductions in their systolic, diastolic, and mean arterial pressures after quercetin treatment [288]. A study was conducted to investigate the effects of quercetin supplementation on blood pressure and lipid metabolism in a group of ninety-three overweight or obese individuals at risk of cardiovascular disease. The participants were randomly assigned to receive 150 mg of quercetin per day in a double-blinded, placebo-controlled crossover trial. The research indicated that quercetin resulted in a reduction in systolic blood pressure (SBP) by 2.6 mmHg across the entire study group, by 2.9 mmHg among the hypertensive participants, and by 3.7 mmHg in the subgroup of younger adults aged 25–50 years compared with the placebo. In summary, the findings suggest that quercetin supplementation may lower the SBP and plasma-oxidized LDL concentrations in overweight individuals at high risk of cardiovascular disease [74]. The research examined the impact of quercetin consumption on cardiovascular risk factors in women diagnosed with type 2 diabetes. A double-blind, randomized clinical trial was carried out involving 72 women who were randomly assigned to either the quercetin group or the placebo group. Participants in the quercetin group received a daily 500 mg capsule. The findings revealed that quercetin intake led to a significant reduction in systolic blood pressure compared with the placebo [289]. A recent study discovered that consuming quercetin-rich supplements derived from onion peel extract had a positive impact on the health of male smokers. The study involved randomly assigning participants to take either a placebo (n = 43) or 100 mg quercetin capsule daily (n = 49). The findings revealed that the quercetin supplementation led to a significant reduction in total cholesterol and LDL-cholesterol levels whereas the placebo did not produce the same results. Additionally, both the placebo and quercetin groups experienced increases in HDL cholesterol, with the increase being notably more pronounced in the quercetin group. Moreover, the quercetin group showed substantial decreases in systolic and diastolic blood pressure. The study suggests that the daily consumption of quercetin-rich supplementation from onion peel extract may improve the blood lipid profiles, glucose levels, and blood pressure, potentially lowering cardiovascular risk [290]. A study was carried out to examine the impact of the daily consumption of quercetin-rich onions on visceral fat. Seventy healthy Japanese participants with a body mass index (BMI) of 23 to less than 30 were recruited and randomly assigned to either the quercetin-rich onion group or the placebo group in a randomized, double-blind, placebo-controlled parallel study. The results suggest that quercetin-rich onion may be beneficial in preventing obesity and improving liver function [291]. The study aimed to determine the impact of quercetin on adiponectin-mediated insulin sensitivity in patients with polycystic ovary syndrome (PCOS). A total of eighty-four women with PCOS were chosen and randomly divided into two groups: treatment and control. The treatment group received 1 g of quercetin (two 500 mg capsules) daily, while the control group received a placebo. The study found that quercetin led to a slight increase in adiponectin levels by 5.56% and in high molecular weight (HMW) adiponectin by 3.9% compared with the placebo. Additionally, it was observed that quercetin reduced the levels of testosterone and LH [292]. In a study where 84 individuals diagnosed with polycystic ovary syndrome were divided into two groups, one group was given 1 g of quercetin (equivalent to two 500 mg capsules) daily for a period of 12 weeks, while the other group received a placebo. The research revealed that the administration of oral quercetin led to improved metabolic characteristics in patients with this disease [293].

## 8. Interactions of Quercetin with Medications

Quercetin, an important flavonoid, and its implication in health management has been proven, and as a result, interactions with common medications need further investigation. It is crucial to understand how quercetin interacts with common medications to confirm their safe and effective use. One study found that quercetin could modify the pharmacokinetics of warfarin, which is a widely used anticoagulant. In the pretreated group, the C_max_ of WAR was enhanced by 30.43%, AUC_0–∞_ by 62.94%, and t_1/2_ by 10.54%, while decreasing its clearance (Cl) by 41.35% relative to the control. Moreover, in co-administered animals, the C_max_ of WARs increased by 10.98%, AUC_0–∞_ by 20.20%, and t_1/2_ by 8.87%, whereas Cl declined by 16.40%. The QUE impacts the pharmacokinetics of WAR, suggesting that adjustments to the WAR dosage may be necessary following confirmatory clinical studies, particularly for patients with thrombotic disorders [294]. Quercetin inhibits the breast cancer resistance protein (BCRP), changing the pharmacokinetics of the drug sulfasalazine. Pretreatment with quercetin (10 mg/kg) in rats led to a 1.8-fold and 1.5-fold increase in the AUC8h and Cmax of the orally administered sulfasalazine, correspondingly. These outcomes suggest that quercetin acts as an inhibitor of BCRP. In light of the high dietary intake of quercetin and its consumption as a dietary supplement, issuing a carefulness about its food–drug interactions should be considered [295].

## 9. Safety, Optimal Dose, and Toxicity Concerns Associated with High Doses

Natural compounds including flavonoids play a crucial activity in disease management through their diverse mechanisms [296,297,298,299]. While they offer safety at specific doses, caution is warranted, as higher doses can lead to toxicity. It is imperative to ascertain any compound’s optimal and safe dosage before use.

The International Agency for Research on Cancer (IARC) assessed the potential carcinogenic risk of quercetin to humans, and based on the available data at that time, concluded that “quercetin cannot be classified as to its carcinogenicity to humans” [300]. However, the information that the enrolled individuals reported no adverse events is given after the repeated intake of 500 mg quercetin daily for 4–8 weeks [43,301]. After taking a relatively low dose of 150 mg of quercetin daily for six weeks, the hematology, liver and kidney function, and serum electrolyte levels in overweight or obese individuals were evaluated. All of the parameters measured remained within normal ranges [74]. In research studies where the participants were given quercetin or plant extracts containing quercetin glycosides for oral consumption for up to 12 weeks, at dosages ranging from 3 to 1000 mg of quercetin per day, no negative effects associated with the compound were observed. This encompassed the absence of changes in hematology, clinical chemistry, and urinalysis parameters [302,303,304]. The study recruited COPD patients with mild to severe lung disease and supplemented them with either a placebo or quercetin at doses of 500, 1000, or 2000 mg/day in an escalating manner. It was observed that the patients did not experience any severe adverse events related to the study drug based on the blood tests. One of the patients reported mild adverse events including gastroesophageal reflux disease. Quercetin was safely tolerated up to 2000 mg/day [305].

Quercetin is recognized for its safety and tolerability in humans. In 2010, the American Food and Drug Administration (FDA) issued a response letter concerning a GRAS notification related to the use of high-purity quercetin as an ingredient in various food products. The FDA expressed no concerns about the conclusion that high-purity quercetin is GRAS (“Generally Recognized As Safe”) for the specified intended uses [306]. A phase 1 dose–escalation study was conducted to assess the safety of quercetin in 30 untreated patients with chronic HCV infection. Quercetin was found to be safe for all participants in the trial. Moreover, eight patients experienced a “clinically meaningful” decrease of 0.41-log in their viral load. Quercetin was safe at doses of up to 5 g daily [307]. In studies where quercetin or plant extracts containing quercetin glycosides were administered orally to participants for durations of up to 12 weeks, at doses ranging from 3 to 1000 mg of quercetin per day, no adverse effects related to the compound were reported. This included no observed changes in hematology, clinical chemistry, or urinalysis parameters [302,303,304]. No negative effects were observed in both male and female participants after taking single oral doses of quercetin (8.5 mg/kg of body weight per day or 600 mg for an average individual weighing 70 kg) in a study that showed an increase in adenosine levels related to quercetin [308]. In Italy, regulations stipulate that the maximum daily permissible intake of quercetin aglycone in dietary supplements is capped at 200 mg. Additionally, the intake of mixed, no specified flavonoids are limited to 1000 mg per day. This underscores the importance of adhering to safe dosage guidelines for flavonoid consumption [309]. The mean daily intake of flavonoids in European, Australian, and US adult populations has been estimated at 435 mg/day [310].

The optimal dose of quercetin varies based on individual health conditions, the types of diseases, the specific formulation utilized, and planned therapeutic effect. Different dosage ranges have therapeutic implications for different pathogenesis. The recommended optimal dose of quercetin as a dietary supplement is generally up to 1000 mg per day [311].

High doses of quercetin, which are usually considered safe, can raise specific safety concerns in human subjects. During long-term supplementation with 1000 mg per day, some individuals experienced minor side effects including mild headaches, tingling sensations, and nausea in the extremities [312]. Quercetin has been shown to inhibit the hERG potassium channel, which may lead to serious cardiac conditions [313].

## 10. Conclusions and Future Prospective

The current treatment for various pathogenic conditions including chemically synthesized drugs is effective but also leads to adverse effects. However, it is essential to consider alternative remedies to inhibit pathogenesis and overcome adverse effect-related problems effectively. In this regard, natural compounds are crucial in preventing and curing diseases through different mechanisms. Quercetin, a flavonoid derived from other sources, such as plants including seeds, flowers, stems, leaves, and animals, has been proven to have an inhibitory action in various types of pathogeneses. The disease-preventing ability of natural products has been evidenced through hepatoprotective, cardioprotective, neuroprotective, antidiabetic potential, and antimicrobial activities. This compound plays a significant role in cancer prevention by influencing cell signaling molecules including inhibiting angiogenesis, inducing autophagy, apoptosis, cell cycle arrest, and modulating other cell signaling molecules.

This compound plays an important role in managing diseases and can be integrated into public health strategies to prevent various pathogeneses through multiple mechanisms. Its primary function in disease management is attributed to its antioxidant and anti-inflammatory potential. It significantly influences different pathological processes by modulating various biological activities and cell signaling pathways.

Even though the outcomes of the current preclinical studies are very promising, the clinical implications of this compound have been significantly impeded by its poor solubility, leading to a notably diminished bioavailability. The rapid metabolism of this compound is another limitation, along with other factors. Future research should aim at creating quercetin analogs or delivery systems to improve its absorption and effectiveness in the body. Additional studies focusing on combinations with other compounds or drugs should be conducted to enhance its therapeutic effects across various pathologies. As there are limited data available regarding quercetin’s safety and mechanism of action, it is essential for future studies to comprehensively investigate the long-term effects of quercetin, encompassing its effectiveness, safety, and mechanism of action in disease management. Furthermore, it is imperative to conduct clinical trials/studies to explore the maximum benefits of this compound in disease treatment and prevention.

## Figures and Tables

**Table 1:** The role of quercetin in the inhibition of pathogenesis via the reduction in oxidative stress.

Activity	Types of Study	Outcome	Refs.
Antioxidant potential	In vitro, intestinal porcine epithelial cell line 1 cells	Quercetin reduces cell damage by increasing the Nrf2 protein levels and regulating glutathione-related redox balance.	[26]
	In vivo, male Wistar rats	Quercetin decreases the urea, bilirubin, BUN, serum enzymes, creatinine, and MDA levels. This compound increases antioxidant markers such as glutathione, total thiols, and catalase.	[28]
	In vivo, male Sprague-Dawley rats	Quercetin significantly reduced nitric oxide metabolites and superoxide anion production, partially protected blood glutathione, and inhibited plasma malondialdehyde levels.	[29]
	In vivo, boxer subject	Quercetin supplements showed a protective effect against oxidative stress by reducing the amount of MDA. This compound increased the activity levels of the antioxidant enzymes.	[30]
	In vivo, rat model	The amount of glutathione peroxidase and superoxide dismutase in tendon tissue was higher in the high quercetin group. The amount of malondialdehyde in tendon tissue was lower in the high quercetin group.	[31]
	In vitro, pheochromocytoma (PC-12) cells	Quercetin has been shown to have a protective effect against apoptosis induced by oxidative stress.	[32]
	In vitro, retinal pigment epithelial (RPE) cells	This compound demonstrated the remarkable ability to shield RPE cells from oxidative damage.	[33]

**Table 3:** Antidiabetic potential of quercetin.

Activity	Study Types	Dose	Mechanism	Outcomes of the Study	Refs.
Antidiabetic potential	In vivo	50 mg/kg	Glucose levels and lipid profiles, liver and kidney injury marker ↓ Antioxidant enzymes ↑	Treatment with this flavonoid improved elevated serum blood glucose levels and insulin levels. This compound inhibited oxidative stress and tissue injury biomarkers. Structure of pancreatic β-cells improved.	[48]
	In vivo	15 mg/kg	Fasting blood sugar and malondialdehyde ↓ Total antioxidant capacity ↑ The mRNA levels of HSP27, HSP70, HSF-1, and glucose-6-phosphatase ↓ The expression of glucokinase ↑	Quercetin caused an increase in the transcript level of glucokinase and decreased stress proteins.	[50]
	In vivo	25 mg/kg	Maintained pancreatic tissue architecture. Lipid peroxidation ↓ Antioxidant enzyme ↑	Quercetin treatment positively affects pancreatic tissues by directly reducing lipid peroxides.	[51]
	In vivo	20 mg/kg	Fasting blood glucose levels liver and kidney marker enzymes ↓ Antioxidant enzyme ↑	Quercetin ameliorates hyperglycemia and oxidative stress.	[54]
In vivo	50 mg/kg		Improved the neurological morphology of sciatic nerve. Prevented myelin and axonal damage. ROS production levels ↓	Quercetin modulates gut microbiota associated with diabetic peripheral neuropathy. It also modulates levels of ROS production.	[55]
	In vivo	20 mg/kg	Body weight and fasting blood sugar levels ↓ The inflammatory markers ↓ Antioxidant enzyme levels ↑ Kidney tissue architecture maintenance. Fibrosis ↓	The finding demonstrates the antidiabetic, antihyperlipidemic, anti-inflammatory, and reno-protective effects of quercetin. This compound boosted the antioxidant enzyme levels and well-maintained kidney architecture.	[56]

**Table 5:** Neuroprotective potential of quercetin.

	Study Types	Animal Model/Cell Lines	Dose	Outcome	Refs.
Neuroprotective potential	In vivo	Mice model	25 mg/kg	This compound reduces extracellular β-amyloidosis, astrogliosis, tauopathy, and amygdala.	[69]
	In vivo	Wistar rat model	10 mg/kg	Reduces DNA fragmentation and prevents neuronal apoptosis. Attenuates mitochondrial swelling, loss of cristae, and chromatin condensation.	[70]
	In vitro	Pc12	100–100 µM	Quercetin protected the PC12 cells in a dependent way.	[71]
	In vivo	Mice model	500 mg/kg	Quercetin can increase apoE levels and reduce the insoluble Aβ levels.	[72]

**Table 7:** Anticancer effects of quercetin via the intonation of cell signaling molecules.

Pathways	Cancer	In Vitro/In Vivo	Dose	Mechanism	Outcome	Refs.
PTEN/PI3K/AKT	Breast	MCF-7	0, 20, 40, 60, 80, and 100 μmol/L	p-PI3K and p-AKT ↓ and PTEN ↑	A high concentration of quercetin decreased the levels of p-AKT and p-PI3K protein. Quercetin enhanced the expression and distribution of PTEN protein.	[197]
Cell cycle	Breast	MDA-MB-231	20 µM	Cell cycle arrest	Cell cycle arrest at S and G2/M phase caused by quercetin.	[198]
Apoptosis	Oral	SAS	40 µM	Induction of apoptosis	Quercetin-induced apoptosis of cells and increased the activities of caspases.	[199]
Angiogenesis	Colorectal	HT-29 cells	5 and 10 µg/mL	VEGF-A VEGFR-2 ↓	Quercetin decreased the VEGF-A protein expression.	[200]
NF-κB p65	Colorectal	HT-29 cells	5 and 10 µg/mL	NF-κB p65 ↓	NF-κB p65 protein expression was decreased by quercetin.	[200]
Invasion and angiogenesis	Esophageal	Eca109	5 μg/mL or 10 μg/mL	VEGF-A, MMP9, and MMP2 ↓	Quercetin decreased MMP2, MMP9, and VEGF-A and suppressed the invasion and migration.	[201]
Cell cycle	Breast	T47D	50 µM	Cells in G2/M phase and the G2/M cells ↑	Quercetin increased accumulation of the cells in the G2/M phase, and the G2/M.	[202]
Apoptosis	Breast	T47D	50 µM	Induction of apoptosis	The number of apoptotic cells increased by quercetin and a combination of doxorubicin.	[202]
Apoptosis	Oral	D10B and YD38	25 μM and 50 μM	Induction of apoptosis	Quercetin causes apoptotic cell death cells.	[203]
P53	Breast	MDA-MB-453	100 µM	P53 ↑	p53 expression was activated by quercetin.	[204]
Autophagy	Brain	U373MG	25, 50, and 75 µM	Induction of autophagy	Induced autophagy.	[205]
EGFR pathway	Prostate	Rats	200 mg/kg	EGFR ↓	Quercetin prevents cancer progression via inhibition of EGFR pathway.	[206]
JNK/ERK MAPK pathway	Leukemia	Mice	120 mg/kg	JNK/ERK MAPK pathway ↓	Activation of the death signaling molecules such as phosphorylated forms of ERK and JNK MAPK indicates quercetin.	[207]
Calcineurin/NFAT pathway	Breast	Mice	34 mg/kg	Calcineurin/NFAT pathway ↓	Quercetin inhibited tumor calcineurin activities. Effects of this compound on calcineurin/NFAT pathway was noticed by decreased subcellular levels of NFATc3.	[208]
STAT3 pathway	Pancreas	PATU-8988 and PANC-1 cells	0, 20, 40, and 80 µM	STAT3, MMP2, MMP7↓	Quercetin inhibits activation of p-STAT3.	[209]

**Table 9:** Synergistic effects of quercetin with drug compounds/drugs.

Activity	Drugs	Study Types	Outcome	Refs
Antibacterial effects	Colistin, amikacin, and meropenem	In vitro	The combinations of quercetin + colistin and quercetin + amikacin showed synergistic activity on the ColR-Ab strains. The combinations of quercetin + colistin and quercetin + amikacin induced membrane damage.	[234]
	Levofloxacin, amikacin ceftriaxone, tobramycin, gentamycin	In vitro	This compound affected the biofilm cell viability and biofilm formation.	[235]
	Florfenicol	In vitro and in vivo	This compound reduced the bacterial load in the many organ tissues of *Cyprinus carpio* compared with the florfenicol group. The viability decreased over time during the combined treatment with quercetin and florfenicol. Furthermore, the synergistic effect was established by the bacterial growth curve.	[236]
	Ampicillin, cephradine, ceftriaxone, imipenem, and methicillin	In vitro	Quercetin alone showed an additive effect with antibiotics. Quercetin as well as other compounds with antibiotics showed synergism.	[237]
	Amoxicillin	in vitro	Quercetin has a proven synergistic effect with amoxicillin against ARS.	[238]
Anti-inflammatory effects	Catechin	In vitro	LPS-stimulated increase in proinflammatory molecules decreased by combined quercetin treatment with catechin.	[239]
	Resveratrol	In vivo	The combination of quercetin as well as resveratrol suppressed obesity and connected inflammation.	[240]
	Curcumin	In vivo	Edema and lymphocyte infiltration was decreased by both curcumin and quercetin. Additionally, both flavonoids reduced NO formation and MDA and restored antioxidant enzymes.	[241]
	Curcumin	In vivo	Pretreatment with curcumin + quercetin synergistically restored the neuropathic dysfunction and oxidative levels.	[242]
Cardioprotective effects	α-Tocopherol	In vivo	Cardioprotective effects of quercetin and α-tocopherol were noted via scavenging free radicals and improving antioxidants.	[243]
	Sitagliptin	In vivo	The level of cholesterol, LDL, and TG restored through the combination of quercetin and sitagliptin.	[244]
Neuroprotective effects	Epicatechin	In vivo	Oral administration of epicatechin plus quercetin reduced hypoxic-ischemic brain damage.	[245]
	*α*-Lipoic acid	In vivo	α-Lipoic acid and quercetin accompanied each other in defending the brain against oxidative stress.	[246]
Antidiabetic effects	Metformin	In vivo	A combination of metformin and quercetin showed a more significant antidiabetic outcome than either drug.	[247]
	Quinic acid	In vivo	Quercetin as well as the quinic acid-treated groups downregulated oxidative stress and hyperglycemia.	[248]

## Data Availability

No new data were created or analyzed in this study.

## Abbreviations

GSH	Glutathione
MDA	Malondialdehyde
SOD	Superoxide dismutase
CAT	Catalase
ROS	Reactive oxygen species
COX-2	Cyclooxygenase-2
TNF-α	Tumor necrosis factor-α
IL	Interleukin
MAPK	Mitogen-activated protein kinase
LPS	Lipopolysaccharide
IBD	Inflammatory bowel disease
CAD	Coronary artery disease
COPD	Chronic obstructive pulmonary disease
BMI	Body mass index
JNK	c-Jun N-terminal kinase
DOX	Doxorubicin
ASP	Aspartate aminotransferase
IARC	International Agency for Research on Cancer
EFGR	Epidermal growth factor receptor
MMPs	Matrix metalloproteinases
BW	Body weight
HUVECs	Human umbilical vein endothelial cells
MPO	Myeloperoxidase
AuNPs	Gold nanoparticles
MIC	Minimum inhibitory concentration
PCOS	Polycystic ovary syndrome
NPs	Nanoparticles
